# Distinct Stromal and Immune Features Collectively Contribute to Long-Term Survival in Pancreatic Cancer

**DOI:** 10.3389/fimmu.2021.643529

**Published:** 2021-02-19

**Authors:** Hassan Sadozai, Animesh Acharjee, Serenella Eppenberger-Castori, Beat Gloor, Thomas Gruber, Mirjam Schenk, Eva Karamitopoulou

**Affiliations:** ^1^Center for Sport, Exercise and Life Sciences, Coventry University, Coventry, United Kingdom; ^2^College of Medical and Dental Sciences, Centre for Computational Biology, Institute of Cancer and Genomic Sciences, University of Birmingham, Birmingham, United Kingdom; ^3^Institute of Translational Medicine, University Hospitals Birmingham National Health Service, Foundation Trust, Birmingham, United Kingdom; ^4^National Institute for Health Research Surgical Reconstruction and Microbiology Research Centre, University Hospital Birmingham, Birmingham, United Kingdom; ^5^Institute of Pathology, University Hospital Basel, Basel, Switzerland; ^6^Department of Visceral Surgery, Insel University Hospital, University of Bern, Bern, Switzerland; ^7^Institute of Pathology, University of Bern, Bern, Switzerland

**Keywords:** PDAC–pancreatic ductal adenocarcinoma, long term survival, tumor associated macrophage (TAM), cancer associated fibroblast (CAF), tumor microenvironment (TME)

## Abstract

**Background:** The aggressive biology and treatment refractory nature of pancreatic ductal adenocarcinoma (PDAC) significantly limits long-term survival. Examining the tumor microenvironment (TME) of long-term survivors (LTS) of PDAC offers the potential of unveiling novel biological insights and therapeutic targets.

**Methods:** We performed an integrated approach involving immunophenotyping, stromal scoring and histomorphological profiling of a cohort of 112 PDAC-cases, including 25 long-term survivors (LTSs, OS ≥ 60 months). Mutational frequencies were assessed using targeted next generation sequencing. Finally, we validated our findings *in silico* using an external cohort of microarray data from PDAC patients.

**Results:** LTS cases exhibit a largely quiescent population of cancer-associated fibroblasts (CAFs). Immune profiling revealed key differences between LTS and NON-LTS cases in the intratumoral and stromal compartments. In both compartments, LTS cases exhibit a T cell inflamed profile with higher density of CD3^+^ T cells, CD4^+^ T cells, iNOS^+^ leukocytes and strikingly diminished numbers of CD68^+^ total macrophages, CD163^+^ (M2) macrophages and FOXP3^+^ Tregs. A large proportion of LTS cases exhibited tertiary lymphoid tissue (TLT) formation, which has been observed to be a positive prognostic marker in a number of tumor types. Using a Random-Forest variable selection approach, we identified the density of stromal iNOS^+^ cells and CD68^+^ cells as strong positive and negative prognostic variables, respectively. In an external cohort, computational cell-type deconvolution revealed a higher abundance of T cells, B lymphocytes and dendritic cells (DCs) in patients with long-term OS compared to short-term survivors. Thus, *in silico* profiling of long-term survivors in an external cohort, strongly corroborated the T cell-inflamed TME observed in our LTS group.

**Conclusions:** Collectively, our findings highlight the prognostic importance of TME profiles in PDAC, underlining the crucial role of tumor associated macrophages (TAMs) and the potential interdependence between immunosuppressive TAMs and activated CAFs in pancreatic cancer. Additionally, our data has potential for precision medicine and patient stratification. Patients with a T cell inflamed TME might derive benefit from agonistic T cell antibodies (e.g., OX40 or CD137 agonists). Alternately, patients with activated CAFs and high infiltration of immunosuppressive TAMs are highly likely to exhibit therapeutic responses to macrophage targeted drugs (e.g., anti-CSF1R) and anti-CAF agents.

## Introduction

Pancreatic ductal adenocarcinoma (PDAC) is an exceptionally lethal malignancy with a 5-years survival of <10% and is projected to become the second-leading cause of cancer-associated mortality by 2030 (1, 2). Recent advances in surgical techniques and multimodal management have improved 5-years overall survival following resection with curative intent (3–6). Nevertheless, the rates of tumor recurrence remain high, limiting the quality of life and long-term survival of patients with PDAC (4). Several factors have been associated with diminished overall survival (OS) in pancreatic cancer including tumor stage, lymph node metastases and involvement of the resection margins (7, 8). However, the aforementioned features are simply indicative of a highly aggressive tumor biology and do not shed mechanistic insight on disease progression. Furthermore, unlike breast or colon cancer, molecular subtyping in PDAC is subject to further study and does not currently inform clinical decision-making (9). Molecular profiling has identified two to five PDAC-subtypes and transcriptional analyses have correlated molecular subtypes to microenvironmental and histomorphologic features (9–14).

It is currently well-established that the tumor microenvironment (TME) of PDAC poses a significant barrier to conventional and immune-modulating cancer therapies (15, 16). PDAC patients generally display a distinct TME marked by a significant stromal compartment comprised of cancer-associated fibroblasts, inflammatory cytokines and multiple populations of immunosuppressive leukocytes in particular, tumor associated macrophages (TAMs) (15, 17). While shown to be effective in a number of tumor types, only a rare subset of PDAC patients (~1%) with high microsatellite instability (MSI-high) derive clinical benefit from anti-PD-1 checkpoint inhibitors (15). Although a number of reports have attempted to elucidate the heterogeneous cellular landscape of the TME in pancreatic cancer (18–22), there is currently an unmet need for defining novel biomarkers and treatments for PDAC. Thus, a more comprehensive assessment of TME is warranted and this might reveal key immune, stromal and tumor-intrinsic features, which are essential for disease progression in PDAC and have prognostic or therapeutic value. Moreover, examining the TME of long-term survivors (LTS) of PDAC might unveil novel biological insights and therapeutic targets.

In this study, we performed an integrated histomorphological, immunological and stromal phenotyping of PDAC tissues from a clinically rare subgroup of patients with long-term survival (LTS, OS ≥ 60 months) and compared them to all other cases (NON-LTS) in our cohort of 112 patients, to demonstrate that long-term survival in pancreatic cancer is associated with distinct stromal and immunological profiles. Finally, we validated our immunological findings *in silico* using gene expression data from an external cohort of PDAC samples.

## Methods

### Patients and Tissues

Overall, 112 cases of surgically resected PDACs, stage I-III were available for this study in ngTMA® format (see Supplementary Materials for TMA construction details). This cohort included 25 long-term survivors (LTS, OS ≥ 60 months) and 87 cases with OS <60 months (NON-LTS). Patients were selected on the base of tissue availability and accessibility to full follow-up information and overall survival. Patient related information can be viewed in the Supplementary Table 1. All patients provided written informed consent for inclusion in this study and the study was approved by the Ethics Committee of the canton Bern (KEK Nr 200/14).

### Assessment of Tumor Budding, Gland-Forming Component, and TLT

Tumor budding was evaluated as previously described (22). The percentage of gland-forming component was assessed using H&E stained whole tissue slides by a trained pathologist. Cases were evaluated as present or absent for TLT based on staining with H&E as well as the patterns of CD3^+^T cells and CD20^+^B lymphocytes (for further details, see Supplementary Materials section).

### Stromal Subtyping and Evaluation

Evaluation of the αSMA was undertaken by assessing the intensity of the staining in each tumor core which ranked from 0 to 3 (0: negative, 1: weak, 2: moderate, and 3: strong) and the percentage of area stained. H-Scores were then calculated for each core by multiplying intensity score by the percentage of core staining and a median H-score was calculated for all cores from each patient. The digitalized slides stained for αSMA were evaluated in parallel with their corresponding H&E-stained slides to ensure smooth-muscle fibers of the duodenal wall were not mistaken for stromal reaction. Collagen quantification was assessed by using Masson‘s Trichrome staining (Collagen stained blue) according to the intensity of the staining in the tumor core which ranked from 0 to 3 (0: negative, 1: weak, 2: moderate, and 3: strong) and the percentage of area stained. H-scores were calculated for each core by multiplying the intensity score by the percentage of core staining. An average H-score was calculated for all cores from each patient. Average H-scores for αSMA and Collagen were dichotomized through the median into low and high scores and cases were assigned accordingly into the corresponding categories as described in the results section.

### Immunohistochemistry

ngTMAs were sectioned at 3 μm, de-waxed and re-hydrated in dH2O. They were double stained immunohistochemically for Pancytokeratin (1:400, cytokeratin LMW, clone AE1/AE3, Dako M3515) and each of the following: CD3 (1:400, clone SP7, Abcam ab16669), CD4 (1:100, clone CD4/4B12, Dako M7310), CD8 (1:100, clone C8/144B, Dako M7103), CD20 (1:100, clone L26, Dako M0755), CD68 (1:100, clone KP1, Dako M0814), DC-LAMP (1:100, CD208/DC-LAMP PA, Sino Biological, 10527-H08H), iNOS (1:100, PAb, Thermo Fisher Scientific PA3-030A), CD163 (1:100, clone 10D6, Novocastra NCL-CD163), FOXP3 (1:100, clone 236A/E7, Abcam ab20034), and αSMA (1:100, clone). Antigen retrieval was performed with Tris-HCl, pH 9 for 30 min at 95°C. Antibody testing and staining protocols have been established and staining was performed by an automated Leica Bond RX System (Leica Bond RX, Leica Biosystems, Muttenz, Switzerland) with the Bond Polymer Refine Kit (with DAB as chromogen) and Bond Polymer Refine Red Detection Kit for the double staining (Leica Biosystems, Newcastle, UK).

### Normalization and Scoring of the Immune Cell Infiltrates

All slides were digitalized with their corresponding H&E-stained slides (Aperio Image Scope, Version 12.4.0.5043) and evaluated by virtual microscopy using the Case Viewer software (Case Viewer 3DHISTECH_Ltd Version 2.2.0.85100). Immune cells in the intratumoral (IT) and stromal (S) compartments were enumerated separately and normalized per unit area as cells or counts/mm^2^. As a single TMA core may contain various degrees of non-tumor vs. tumor content, the percentage of non-tumor and tumor tissue per core was recorded. Cell counts were normalized for tumor and non-tumor content of each core. For each immune cell population, the average counts across all TMA cores of the same patient were used for further analysis. Evaluation was performed by two independent pathologists blinded to clinical parameters.

### Next Generation Sequencing and Data Analysis

Libraries preparation using the Ion AmpliSeq™ Cancer Hotspot Panel v2 panel (Thermo Fisher Scientific) were performed following the manufacturer's instructions and as previously described (23). The Ion AmpliSeq™ Cancer Hotspot Panel v2 panel was designed to allow amplification-based capture and sequencing of nearly 2800 COSMIC mutations from 50 oncogenes and tumor suppressor genes. The NGS was performed on an Ion S5^TM^ system (Thermo Fisher Scientific). Finally, all candidate mutations were manually reviewed using the Integrative Genomics Viewer (24).

### Gene Expression Dataset

The expression matrix and associated metadata from the Puleo cohort (12) were obtained from ArrayExpress (accession number E-MTAB-6134). Expression data was downloaded in pre-processed form and no further transformation or normalization was applied. Probe annotation was downloaded from GEO for [HG-U219] Affymetrix Human Genome U219 Array (platform ID GPL13667). To ensure most up-to-date gene symbol nomenclature, we re-annotated probes with relevant Entrez gene IDs in the annotation file and assigned gene symbols using “org.Hs.eg.db” package in BioConductor (https://bioconductor.org/packages/release/data/annotation/html/org.Hs.eg.db.html). In cases where single probe had multiple gene ID assignments, the probe was replicated to the number of gene IDs assigned to it. To reduce probe-level to gene-level expression, intensity values were averaged per gene by calculating mean value for all the probes with the same gene symbol. The resulting gene-level expression values were used for all downstream analysis except for differential expression, which was performed at the probe-level.

### Gene Signature Scoring

The TLS gene signature was first reported by Coppola et al. (25) and consists of the following 12 chemokine genes: *CCL2, CCL3, CCL4, CCL5, CCL8, CCL18, CCL19, CCL21, CXCL9, CXCL10, CXCL11, CXCL13*. The normalized signature scores for a given gene signature were calculated for each sample using *singscore* package in Bioconductor (26), following established user guidelines and gene-level values for Puleo datasets.

### Cell-Type Deconvolution

The MCP-counter algorithm was performed using “immunedeconv” package in R, a uniform access-tool for multiple cell-type deconvolution methods (27). Gene-level values from Puleo dataset were reverse-transformed by exponentiating base 2 to the power of each expression value according to package user guide suggestion. In all cases “tumor” flag was set to “TRUE” and “arrays” flag was set to ‘TRUE‘ for Puleo dataset.

Cell type abundance scores for each of the groups (LTS vs. STS) were visualized as box plots. Significance of difference between groups was assessed using Mann-Whitney *U* test.

### Random Forest Machine Learning and Immune Variable Selection

We used Random Forest (RF) (28), a machine learning ensemble method to identify the immune cell type, the density of which can best predict LTS vs. NON-LTS status. RF uses a bootstrapping method that generates random samples from the dataset with replacement. Those samples are divided into training (Two thirds of the sample set) and testing samples (One third of the sample set). The testing samples are also termed “out-of-bag” (OOB) sample and used for the prediction performance of the model. RF needs to use the number of trees (ntree) and number of variables (either IT or S cell types) randomly sampled as candidates at each split (mtry), and these parameters need to be defined. We used ntree = 500 and mtry = square root of variables in our models.

We used two strategies to select best markers that are associated with survival. First, we iteratively fitted random forests, at each iteration building a new forest after discarding 20% of the variables with the smallest variable importance. The selected set of variables is used as a predictor to fit the model to check the OOB error rate. This recursive feature elimination method procedure is done iteratively using the varSelRF function from the *varSelRF* package in R (28). Second, the selected variables are further refined based on the smallest set of variables with the best AUC values.

### Survival Analysis

Kaplan-Meier plots and log-rank tests were performed to determine differences in overall survival between groups. These were plotted and analyzed in the *survminer* package in R. The cut-offs for patient stratification for each survival curve are provided in the figure legends. Multivariate Cox Regression was performed using *survival* package in R accounting for patient sex, tumor stage, and grade.

### Statistical Analysis

Statistical differences between continuous variables or immune cell counts were analyzed using Mann-Whitney *U* tests or Kruskal Wallis tests followed by Dunn's *post-hoc* test. Differences between categorical variables were calculated by means Fisher's exact test or Chi-square tests. Analyses were conducted on Prism (Version 8.3) and R (Version 4.0.0). All tests were two sided and *P*-values were considered significant at *P* < 0.05.

## Results

### Clinical and Pathological Profiles

We integrated immunophenotyping with stromal and histomorphological profiling on surgically resected, pre-treatment, PDAC tissues (Stage I-III) in the format of a next-generation tissue microarray (ngTMA®) as outlined in Supplementary Figure 1. There was no significant association between the survival group (LTS vs. NON-LTS) and any of the following clinical features: gender, age, or tumor size. However, a significant proportion of LTS cases exhibited lower tumor grades, CA19-9 values and AJCC stages compared to NON-LTS patients (Supplementary Table 1).

Molecular profiling of our cohort was conducted via next generation sequencing using Ion AmpliSeq™ Cancer Hotspot Panel v2. A comparison between LTS and NON-LTS cases for frequencies of mutations in the 4 key driver genes *KRAS, TP53, CDK2NA*, and *SMAD4* is shown in Supplementary Table 2. No significant differences between the mutational frequencies of *KRAS, TP53, CDK2NA*, and *SMAD4* were observed between the two survival groups. These findings collectively demonstrate that LTS status is not associated with a markedly altered profile of PDAC driver mutations.

**Figure 1:**
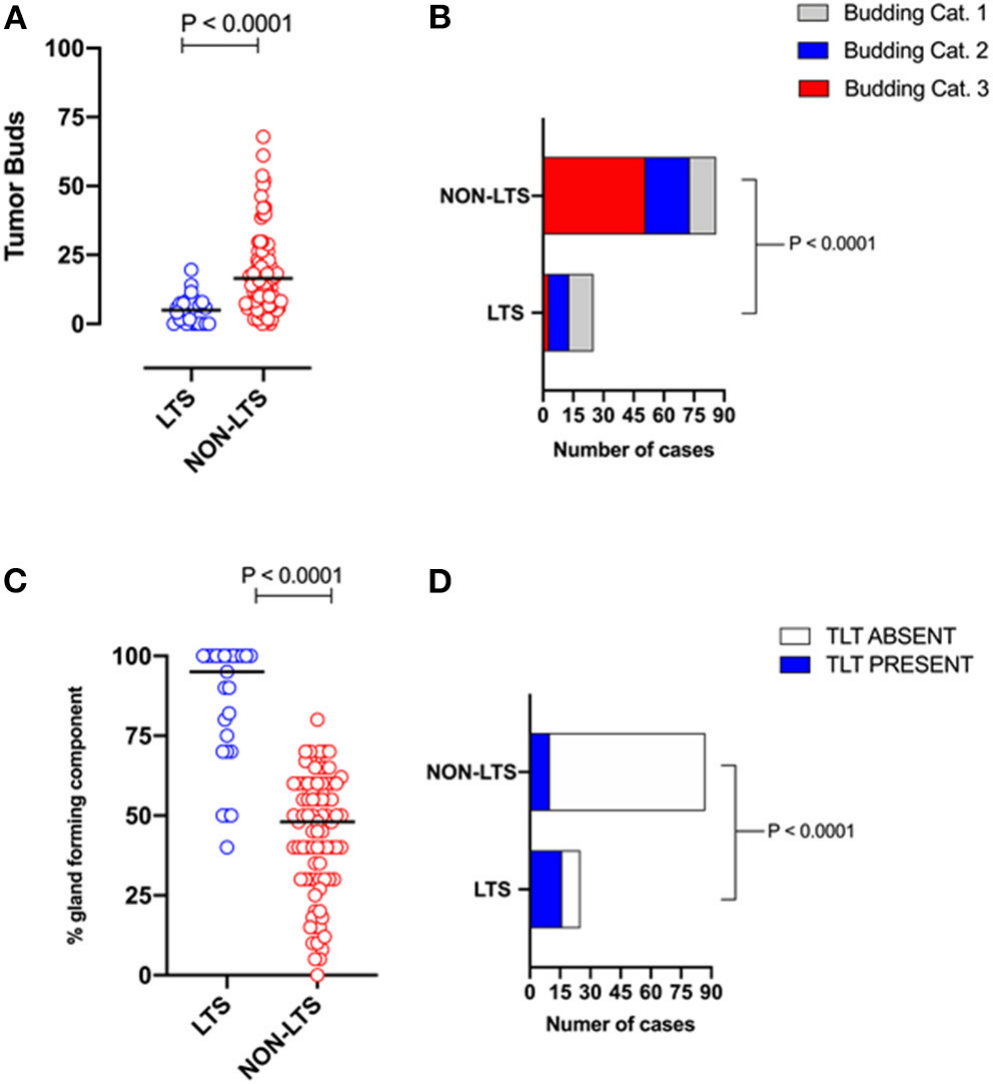
LTS cases exhibit distinct histopathological characteristics. **(A)** Scatterplot depicting the distribution of tumor buds in LTS vs. NON-LTS cases. Mann-Whitney *U*-test was used to determine differences between groups. Each symbol denotes an individual patient. The bars denote median values for each group. **(B)** Bar-graphs showing the distribution of tumor budding categories in LTS and NON-LTS patients. Statistical comparisons were performed using Chi-Square test. **(C)** Scatterplot depicting the distribution of gland-forming component (as a percentage) in LTS vs. NON-LTS cases. Mann-Whitney *U*-test was used to determine differences between groups. Each symbol denotes an individual patient. The bars denote median values for each group. **(D)** Bar-graphs depicting the number of cases exhibiting presence or absence of tertiary lymphoid tissue (TLT) in LTS and NON-LTS patients. Statistical comparisons were performed using Fisher's exact test.

However, LTS cases exhibit distinct histomorphological differences from NON-LTS patients (Figure 1 and Table 1). A comparison of LTSs vs. non-LTSs demonstrated significant differences with respect to the number of tumor buds as well as tumor budding category (Figures 1A,B). While high-grade tumor budding (Category 3) cases represented only 12% of the LTS cohort, they comprised 60% of the NON-LTS cases (*P* < 0.0001). A key morphological characteristic which has recently been shown to have positive prognostic significance for PDAC patients is the percentage of the gland-forming component (13). A statistically significant difference was observed between LTS and NON-LTS cases with respect to this important morphological feature (*P* < 0.0001, Figure 1C). Finally, we performed histological assessment for the presence of tertiary lymphoid tissue (TLT), also termed tertiary lymphoid structures (TLS). TLS are ectopic lymphoid aggregates found in chronic inflammatory conditions and in many solid tumors and are observed to be a positive prognostic factor in PDAC (29). In the LTS group, a significantly higher (*P* < 0.0001) proportion of cases displayed TLT formation compared to the NON-LTS group (64 vs. 12%, respectively) as shown in Figure 1D. Representative images of TLT formation in PDAC are shown in Supplementary Figure 2A. The presence of TLT was associated with improved survival in this cohort (*P* < 0.0001; Supplementary Figure 2B).

**Table 1:** Tumor budding, percentage of gland-forming component and presence of TLT across survival groups in PDAC.

**Parameter**	**LTS (*n* = 25)**	**NON-LTS (*n* = 87)**	***P*-value**
**Tumor budding category**			
3	3 (12%)	52 (60%)	**<0.0001**
2	9 (36%)	22 (25%)	
1	13 (52%)	13 (15%)	
Percentage of gland-forming component median (range)	95 (40–100)	48 (0–80)	**<0.0001**
TLT status			**<0.0001**
*Present*	16 (64%)	10 (12%)	
*Absent*	9 (36%)	77 (88%)	

*Quantitative data presented as group medians with interquartile range (IQR) in brackets. Categorical data are shown number of cases (and as percentages of survival subgroup in brackets). Statistical comparisons were performed using Mann-Whitney U Test, Fisher's Exact Test and Chi-Square test. Bolded values denote significant results*.

Representative images of the differences between LTS and NON-LTS cases in the percentage of gland forming component and tumor budding are shown in Supplementary Figures 3A,B. Thus, taken together, these findings demonstrate that long-term survivors of pancreatic cancer exhibit distinct histomorphological characteristics.

### Stromal Subtyping

Pancreatic stellate cells are the primary subset of cancer-associated fibroblasts (CAFs) in PDAC (30). Upon activation, PSCs express alpha smooth muscle actin (αSMA) and produce factors contributing to disease progression as well as producing extracellular matrix (ECM) proteins such as collagen (30, 31). Using semi-quantitative histological or H-Scores, each case was scored as high or low for αSMA and collagen expression via a median cut-off (32). Based on these classifications, we scored the stromal subtypes as inert (αSMA^low^/collagen^low^), desmoplastic (αSMA^low^/collagen^high^), fibrolytic (αSMA^high^/collagen^low^), and fibrogenic (αSMA^high^/collagen^high^). Representative images for stromal subtypes are shown in Supplementary Figure 4.

**Figure 2:**
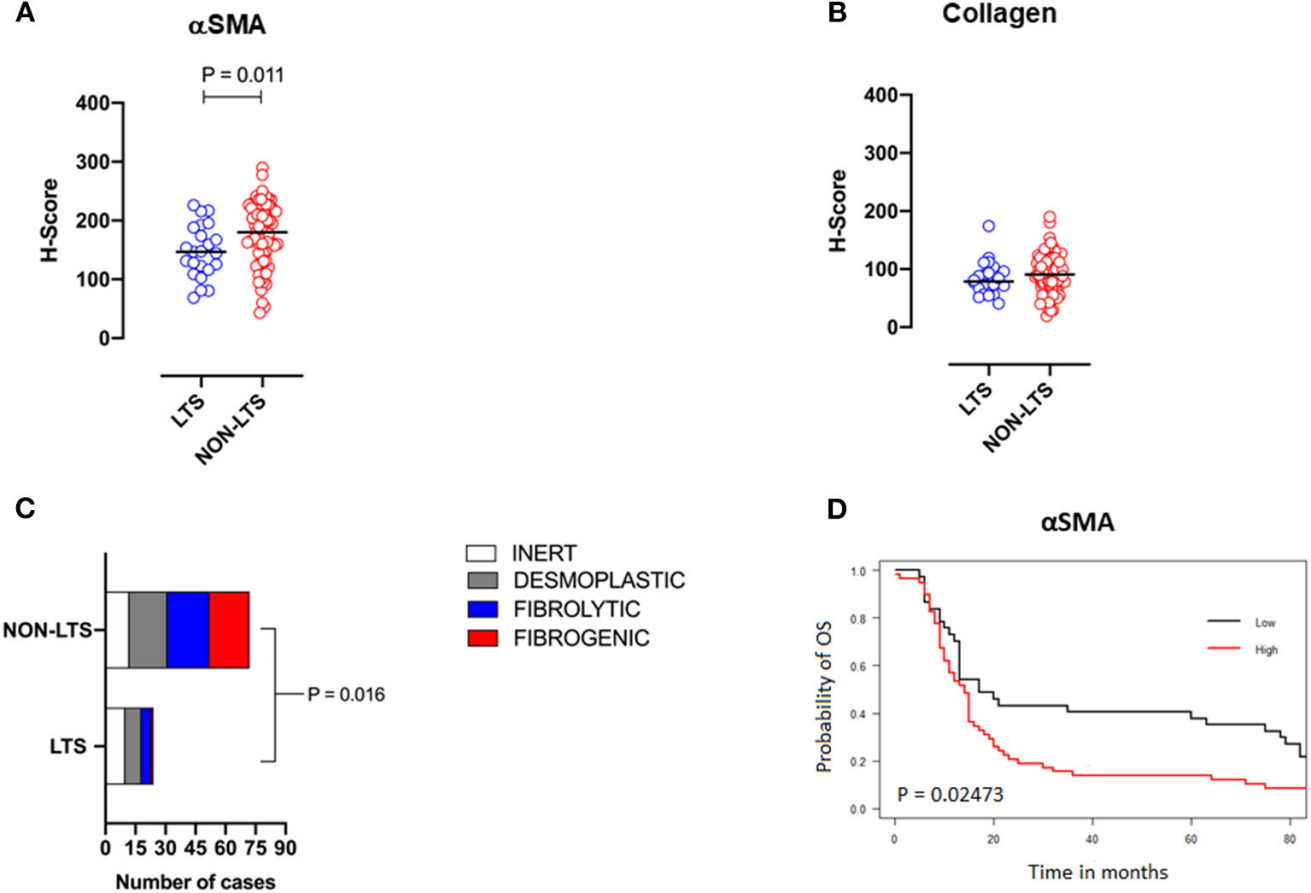
Stromal subtyping of the TME. **(A)** Scatterplots depicting H-Scores for αSMA staining (IHC) in LTS vs. NON-LTS groups. **(B)** Scatterplots depicting H-Scores for Collagen staining (Masson's trichrome) in LTS vs. NON-LTS cases. Mann-Whitney *U* test was used to determine differences between groups. Each symbol denotes an individual patient. The bars denote median values for each group. **(C)** Barplots depicting number of cases belonging to each stromal subtype (inert, desmoplastic, fibrolytic and fibrogenic) in LTS vs. NON-LTS patients. Statistical comparisons were performed using Chi-square test. **(D)** Kaplan-Meier curve showing the correlation of αSMA values with overall survival (OS). Statistical comparisons were performed using log-rank test.

When H-scores for αSMA and collagen were compared between LTS and NON-LTS cases, the expression of αSMA was observed to be significantly higher in long-term survivors (*P* = 0.011, Figure 2A). However, collagen expression levels were comparable between both survival groups (Figure 2B). We subsequently compared the distribution of stromal subtypes between LTS and NON-LTS groups (Figure 2C and Table 2). Here, it was shown that the “inert” and “desmoplastic” subtypes which are αSMA^low^ comprised 75% of the LTS group compared to 43% of the NON-LTS group. Similarly, the most fibrotic phenotype, i.e., the “fibrogenic” subtype constituted only 4% of LTS cases compared to 28% of NON-LTS patients. The association between survival groups and stromal subtype was analyzed by the Chi-square test and found to be statistically significant (*P* = 0.016). Finally, increased levels of αSMA were associated with worse outcome in our cohort demonstrating the importance of the stromal compartment in modulating disease progression (*P* = 0.02473, Figure 2D).

**Table 2:** Features of tumor desmoplasia and stromal subtyping.

**Feature**	**LTS**	**NON-LTS**	***P-*value**
aSMA H-Score	146.60 (68.55–226.10)	180.00 (43.00–290.00)	**0.011**
Collagen H-Score	78.89 (40.89–174.4)	90.63 (19.00–190.00)	0.20
Stromal subtypes			**0.016**
*Inert (aSMA^*low*^/Collagen^*low*^)*	42%	17%	
*Desmoplastic (aSMA^*low*^/Collagen^*high*^)*	33%	26%	
*Fibrolytic (aSMA^*high*^/Collagen^*low*^)*	21%	29%	
*Fibrogenic (aSMA^*high*^/Collagen^*high*^)*	4%	28%	

*Quantitative data presented as group medians with interquartile range (IQR) in brackets. Categorical data are shown as percentages of survival subgroup. Statistical comparisons were performed using Mann-Whitney U Test and Chi-Square test. Bolded values denote significant results*.

**Figure 3:**
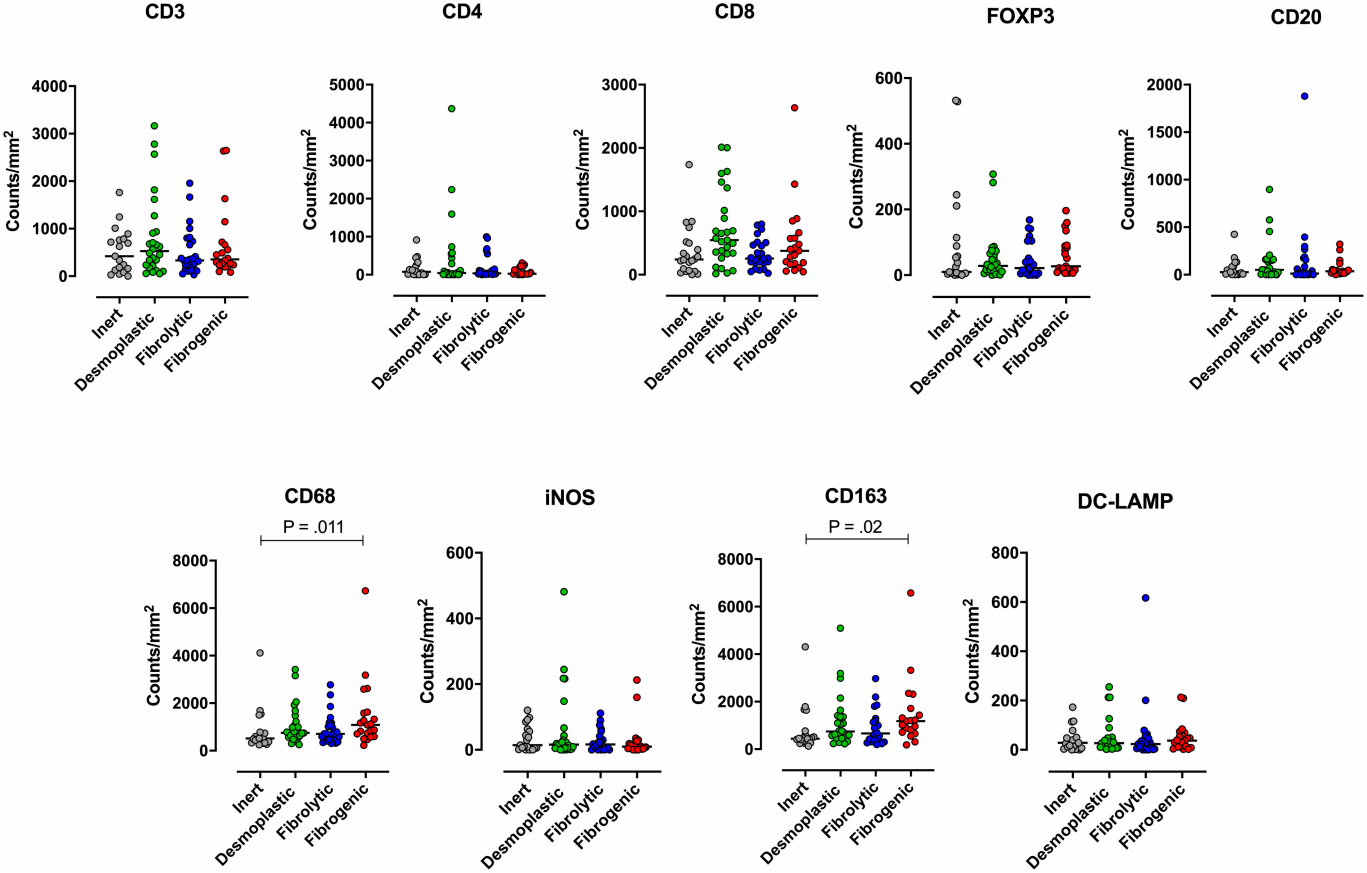
Immune contexture across stromal subtypes. Scatterplots representing the distribution of leukocyte density (counts/mm^2^) in the stromal compartment of PDAC cases exhibiting distinct stromal subtypes (Inert, Desmoplastic, Fibrolytic, Fibrogenic). Each symbol denotes an individual patient. The bars denote median values for each group. Differences between groups were analyzed by Kruskal-Wallis tests followed by Dunn's *post-hoc* test. Multiplicity adjusted *P*-values are reported.

To examine the association between stromal subtypes and immune composition, immune cell densities were compared across all stromal subtypes (Figure 3 and Table 3). We performed immunohistochemistry-based detection and enumeration of nine immune cell subsets; total T cells (CD3), cytotoxic T cells (CD8), helper T cells (CD4), Tregs (FOXP3), B cells (CD20), total macrophages (CD68), M2 macrophages (CD163), iNOS leukocytes (which include M1 macrophages and neutrophils), and mature dendritic cells (DC-LAMP). The counts of CD68^+^ and CD163^+^ (putatively M2) macrophages were significantly higher in cases with “fibrogenic” compared to “inert” stroma (*P* = 0.011 and *P* = 0.02, respectively), revealing a potential biological interdependence between immunosuppressive TAMs and activated CAFs. Taken together, these results demonstrate that LTS patients display a distinct stromal profile marked by largely quiescent PSCs and diminished fibrosis.

**Table 3:** Distribution of stromal (S) leukocyte densities across stromal subtype groups.

	**Inert**		**Desmoplastic**		**Fibrolytic**		**Fibrogenic**		
**Immune cell type**	**Median**	**Range**	**Median**	**Range**	**Median**	**Range**	**Median**	**Range**	***P*-value**
CD3/ mm^2^	420.6	3.25–1,760	526.8	54.27–3,163	333	31.87–1,957	354.1	84.97–2,647	0.71
CD4/mm^2^	77.08	0–912.9	43	0–4,367	36.51	0–995.7	24.8	0–308.7	0.92
CD8/mm^2^	240.7	0–1,736	545.4	16.12–2,013	253.4	24.4–799.1	377	46.17–2,635	0.08
CD20/mm^2^	25.95	0–424.7	52.61	0–5,811	12.38	0–1,879	35.22	0–321.8	0.58
DC-LAMP/mm^2^	28.29	0–173.1	26.97	2.65–255.3	23.59	0–617	37.04	2.53–212.8	0.62
CD68/mm^2^	517.1	244.7–4,116	752.5	253.1–3,422	706.9	309.4–2,777	1084	229.6–6,730	**0.011**
iNOS (M1) /mm^2^	14.17	0–120.3	15.8	0–481.5	16.37	0–111.4	9.66	0–212.8	0.67
CD163 (M2) /mm^2^	443.4	130.9–4,310	743.4	228.6–5,096	662.4	199.6–2971	1184	182.6–6,578	**0.02**
FOXP3 (Tregs) /mm^2^	9.8	0–531.6	27.95	0–307.3	21.27	0–168	26.91	5.74–196.4	0.59

*Statistical comparisons were performed using Kruskal-Wallis test. Bolded values denote significant results*.

### Profiling the Immune Contexture

Subsequently, we examined the immune landscapes of LTS vs. NON-LTS patients. The nine leukocyte subsets assessed across stromal subtypes were analyzed between LTS and NON-LTS cases. Immune cell counts were assessed separately for the intratumoral (IT) and stromal (S) compartments to reveal any compartment-specific differences in the prognostic value of infiltrating immune cells. Representative immunohistochemistry of selected stains for immune cell markers in LTS and NON-LTS-cases is provided in Supplementary Figure 5.

**Figure 4:**
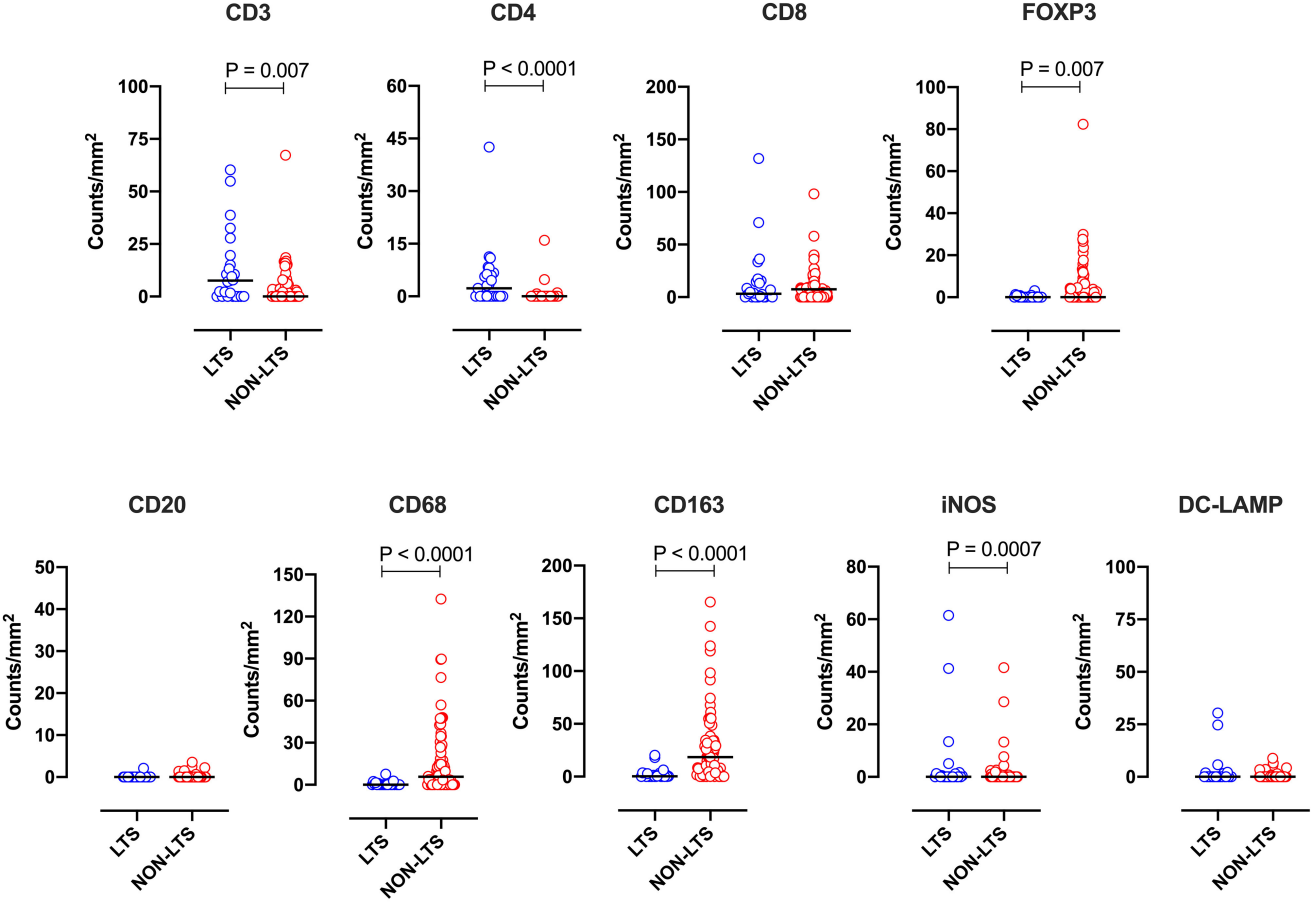
Intratumoral (IT) immune contexture. Scatterplots displaying the distribution of intratumoral leukocyte density (counts/mm^2^) for LTS cases and NON-LTS cases. Each symbol denotes an individual patient. The bars denote median values for each group. Differences between LTS and NON-LTS groups were analyzed using the Mann-Whitney *U* test.

**Figure 5:**
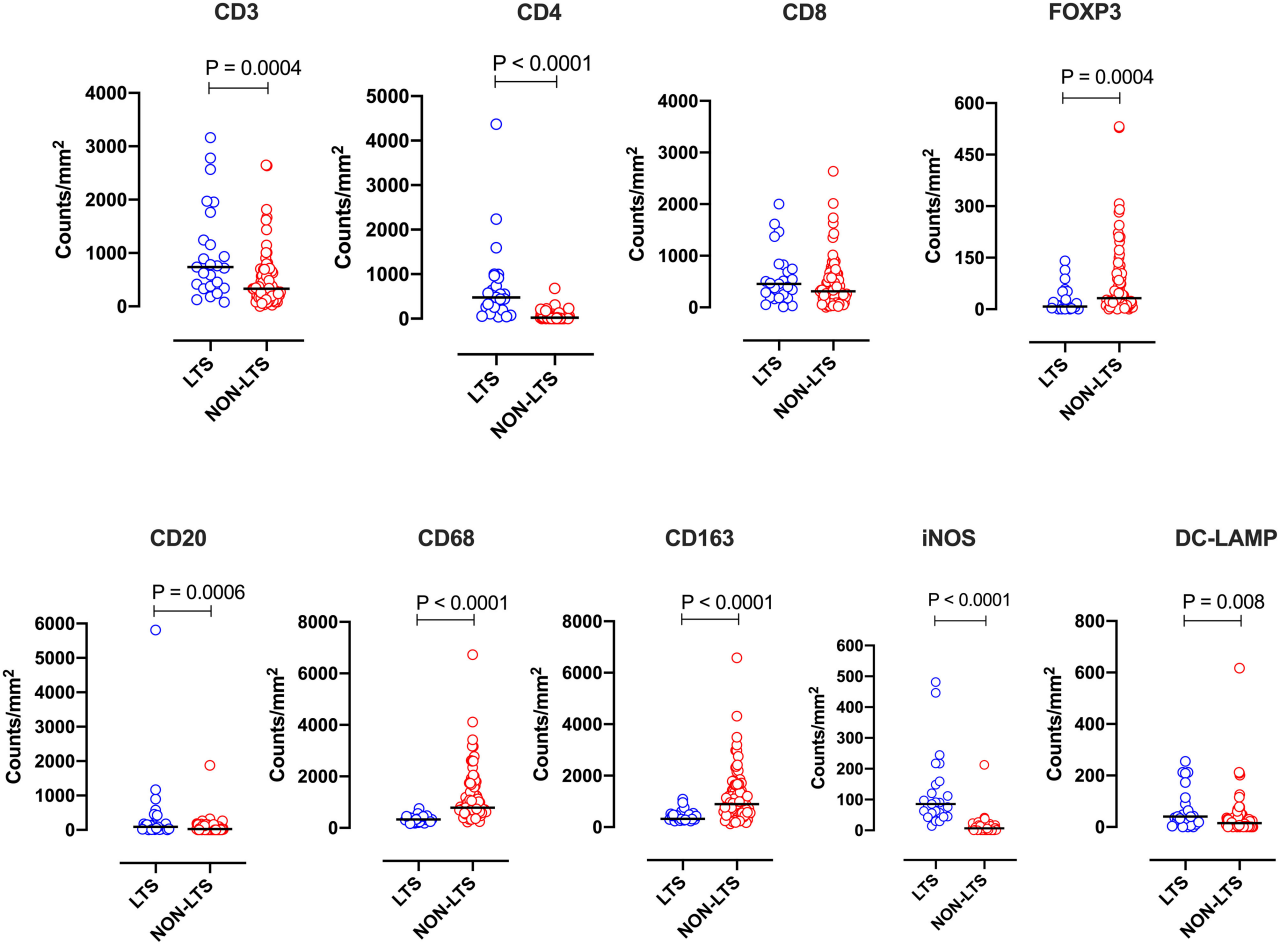
Stromal (S) immune contexture. Scatterplots displaying the distribution of stromal leukocyte density (counts/mm^2^) for LTS cases and NON-LTS cases. Each symbol denotes an individual patient. The bars denote median values for each group. Differences between LTS and NON-LTS groups were analyzed using Mann-Whitney *U* test.

**Figure 6:**
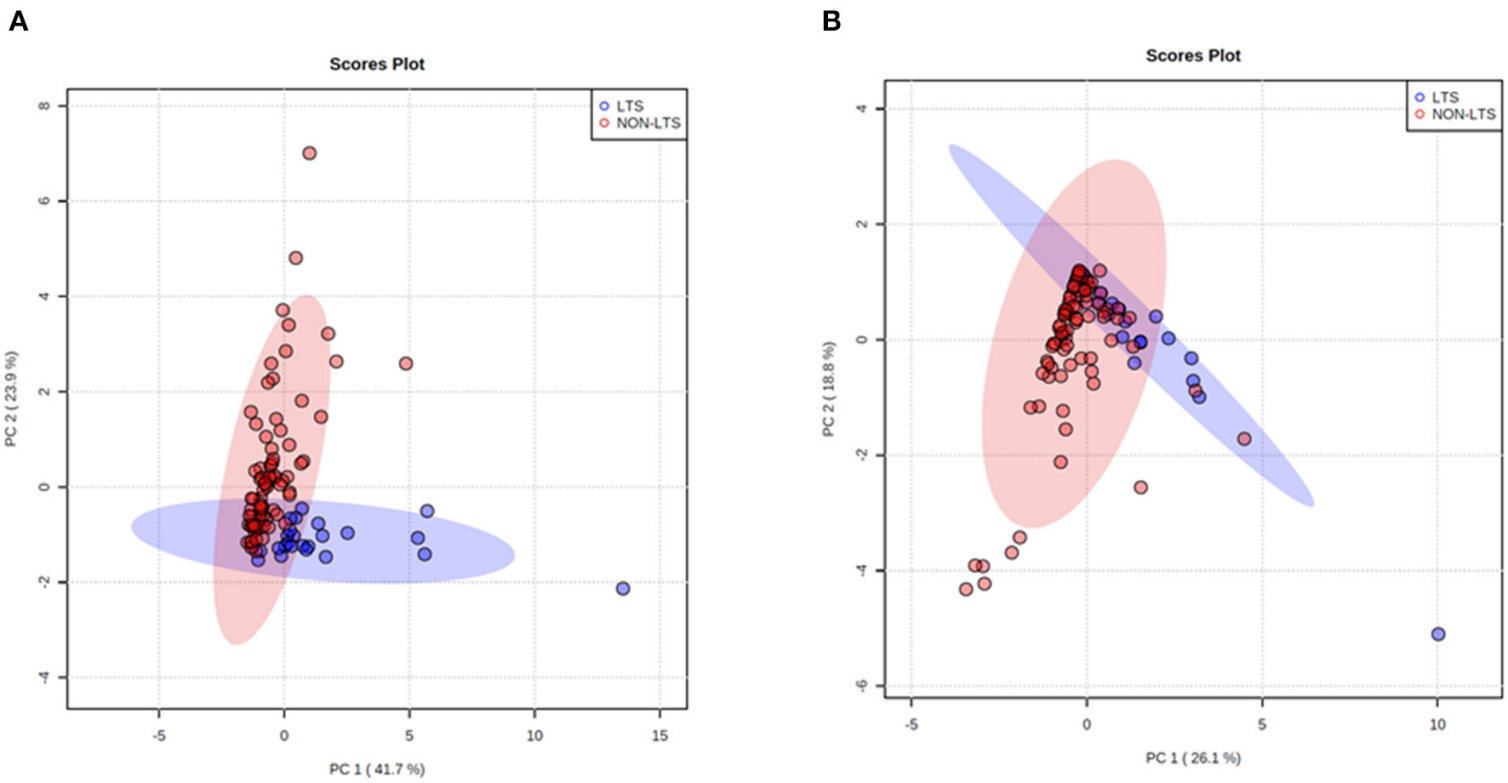
Principal Component Analysis (PCA) demonstrates distinct immune landscape in LTS vs. NON-LTS samples. PCA performed using **(A)** stromal and **(B)** intratumoral immune cell densities show distinct clustering of LTS vs. NON-LTS patient groups. Each symbol denotes an individual patient and 95% confidence ellipses are drawn. The percent variation is explained by first principal component (PC1, x-axis) and the second principal component −2 (PC2, y-axis).

Clear immunological differences were observed between LTS and NON-LTS cases in the IT compartment (Figure 4 and Table 4). Compared to NON-LTS cases, LTS patients exhibit higher densities of total T cells (CD3^+^), CD4^+^ T cells and iNOS^+^ leukocytes (*P* = 0.007, <0.0001 and 0.0007, respectively). On the other hand, LTS patients display significantly lower densities of FOXP3^+^ Tregs, CD68^+^ cells (total macrophages) and CD163^+^ (M2) macrophages than NON-LTS cases (*P* = 0.007, <0.0001, and <0.0001, respectively). No significant differences were observed between LTS and NON-LTS groups in CD8^+^ lymphocytes counts in the IT compartment. Stromal leukocyte densities were not only markedly higher than those in the IT compartment, but also revealed a distinct immune landscape in LTS cases compared to NON-LTS patients (Figure 5 and Table 5). Stromal lymphocyte counts such as CD3^+^ T cells, CD4^+^ helper T cells and CD20^+^ B cells were observed to be higher in the LTS vs. NON-LTS group (*P* = 0.0004, <0.0001, and 0.0006, respectively). On the other hand, FOXP3^+^ Tregs were lower in the LTS group compared to NON-LTS patients (*P* = 0.0004). The myeloid landscape in the stroma was similar to the intratumoral compartment. PDACs from LTS patients displayed significantly higher densities of iNOS^+^ leukocytes (*P* < 0.0001) and DC-LAMP^+^ or “mature” DC (*P* = 0.008) compared to NON-LTS patients. Conversely, stromal densities of CD163^+^ macrophages and CD68^+^ (total) macrophages were significantly lower in LTS vs. NON-LTS cases (*P* < 0.0001, for both cell types). Taken together, these results show that LTS cases exhibit multiple hallmarks of enhanced anti-tumor immunity. Compared to NON-LTS patients, tumors from LTS cases exhibit a T cell inflamed TME with diminished populations of macrophages and Tregs. In order to determine if immune features could distinguish LTS from NON-LTS cases, Principal Component Analysis (PCA), a dimensionality reduction technique was applied, and it was observed that PCA on both IT and stromal immune cell profiles resulted in a distinct separation of LTS from NON-LTS cases (Figures 6A,B). As such, these findings suggest that the improved survival of the LTS cases is associated strongly with an immunologically unique TME.

**Table 4:** Distribution of intra-tumoral (IT) leukocyte densities between LTS and NON-LTS cases.

	**LTS**		**NON-LTS**		
**Immune cell type**	**Median**	**Range**	**Median**	**Range mm^**2**^**	***P*-value**
CD3/mm^2^	7.60	0.00–60.27	0.00	0.00–67.31	**0.007**
CD4/mm^2^	2.310	0.00–42.55	0.00	0.00–16.00	**<0.0001**
CD8/mm^2^	3.19	0.00–131.90	1.740	0.00–98.19	0.28
Foxp3/mm^2^	0.00	0.00–3.19	0.00	82.34	**0.007**
CD20/mm^2^	0.00	0.00–2.12	0.00	0.00–3.53	0.50
CD68/mm^2^	0.00	0.00–7.56	5.80	0.00–132.50	**<0.0001**
CD163/mm^2^	0.34	0.00–20.21	18.43	0.00–165.50	**<0.0001**
iNOS/mm^2^	0.00	0.00–61.44	0.00	0.00–41.56	**0.0007**
DC–LAMP/mm^2^	0.00	0.00–30.37	0.00	0.00–8.85	0.16

*Statistical comparisons were performed using Mann-Whitney U Test. Bolded values denote significant results*.

**Table 5:** Distribution of stromal (S) leukocyte densities between LTS and NON-LTS cases.

	**LTS**		**Non-LTS**		
**Immune cell type**	**Median**	**Range**	**Median**	**Range**	***P*-value**
CD3/mm^2^	735.6	78.89–3163.00	332.40	3.25–2647.00	**0.0004**
CD4/mm^2^	474.50	42.55–4324.00	20.28	0.00–680.90	**<0.0001**
CD8/mm^2^	454.90	10.93–2004.00	309.50	0.00–2635.00	0.24
Foxp3/mm^2^	7.97	0.00–140.80	32.02	0.00–531.90	**0.0004**
CD20/mm^2^	89.25	0.74–5811.00	24.17	0.00–1879.00	**0.0006**
CD68/mm^2^	331.60	169.00–752.50	788.40	229.60–6730.00	**<0.0001**
CD163/mm^2^	319.90	234.60–1085.00	893.60	130.90–6578.00	**<0.0001**
iNOS/mm^2^	85.40	14.17–481.50	6.78	0.00–212.80	**<0.0001**
DC–LAMP/mm^2^	40.76	0.00–255.3	14.82	0.00–617.00	**0.008**

*Statistical comparisons were performed using Mann-Whitney U Test. Bolded values denote significant results*.

### Prognostic Significance of Immune Infiltrates

Given that immune infiltrates could distinguish LTS from NON-LTS cases, we investigated the prognostic significance of leukocyte densities within our cohort. Kaplan-Meier analysis of patients with high and low immune cell infiltration in the stroma (dichotomized by a median cut-off) was performed. Cases with high overall stromal densities of total T cells (CD3^+^) and CD4^+^ T cells displayed significantly improved OS while cases with high densities of stromal FOXP3^+^ Tregs displayed poor OS (Supplementary Figure 6). Patients with high CD8^+^ T cell also displayed improved survival. Moreover, patients included in the high subgroup for stromal densities of CD68^+^ macrophages and M2 (CD163^+^) macrophages had significantly reduced OS while patients with high stromal counts of iNOS^+^ leukocytes exhibited significantly higher OS (Supplementary Figure 6). There were no differences in survival between patients with high vs. low stromal counts of DC (DC-LAMP^+^) and as such, these data are not shown for graphical clarity. Overall, the most significant survival differences between high and low immune infiltration groups were observed for CD4^+^ T cells (*P* = 0.0001), CD68^+^ macrophages (*P* < 0.0001), CD163^+^ macrophages (*P* = 0.0004) and iNOS^+^ leukocytes (*P* < 0.0001).

In order to determine if immune cell infiltration could independently predict OS, we performed multivariate Cox regression analysis including both IT and stromal counts, as distinct variables, in addition to the primary clinical parameters (Table 6). It was observed that along with clinical parameters such as age and UICC stages, stromal densities of total T cells (CD3^+^), CD4^+^ T cells, CD8^+^ T cells, and CD20^+^ B cells were identified as independently predictive of survival. Stromal densities of FOXP3^+^ Tregs and mature DC (DC-LAMP^+^) were also found to be independently prognostic variables in our cohort. In the IT compartment, only CD163^+^ (M2) macrophages were found to be significantly associated with survival in our multivariate cox regression model. All covariates tested in the model are presented in Supplementary Table 3.

**Table 6:** Independent prognostic variables as determined by multivariate Cox regression using data from University of Bern cohort.

**Parameters**	**HR**	**Lower.95**	**Upper.95**	***P*-value**
Age	0.974742	0.952607	0.997392	**0.03**
UICCIB	4.029215	1.037952	15.64096	**0.04**
UICCIIA	7.794558	1.414953	42.9379	**0.02**
UICCIIB	7.257432	2.040314	25.81482	**0.002**
UICCIII	11.08317	2.72406	45.09324	**0.0008**
CD163 IT	1.015146	1.003463	1.026965	**0.01**
CD3 S	1.001003	1.00012	1.001886	**0.02**
CD4 S	0.998119	0.996678	0.999561	**0.01**
CD8 S	0.997823	0.996611	0.999038	**0.0004**
CD20 S	1.001441	1.00033	1.002554	**0.01**
DC-LAMP S	0.995403	0.991113	0.999712	**0.04**
FOXP3 S	1.00403	1.000643	1.007428	**0.02**

*Only statistically significant values are reported. For the full analysis, please see Supplementary Table 3. Bolded values denote significant results*.

**Figure 7:**
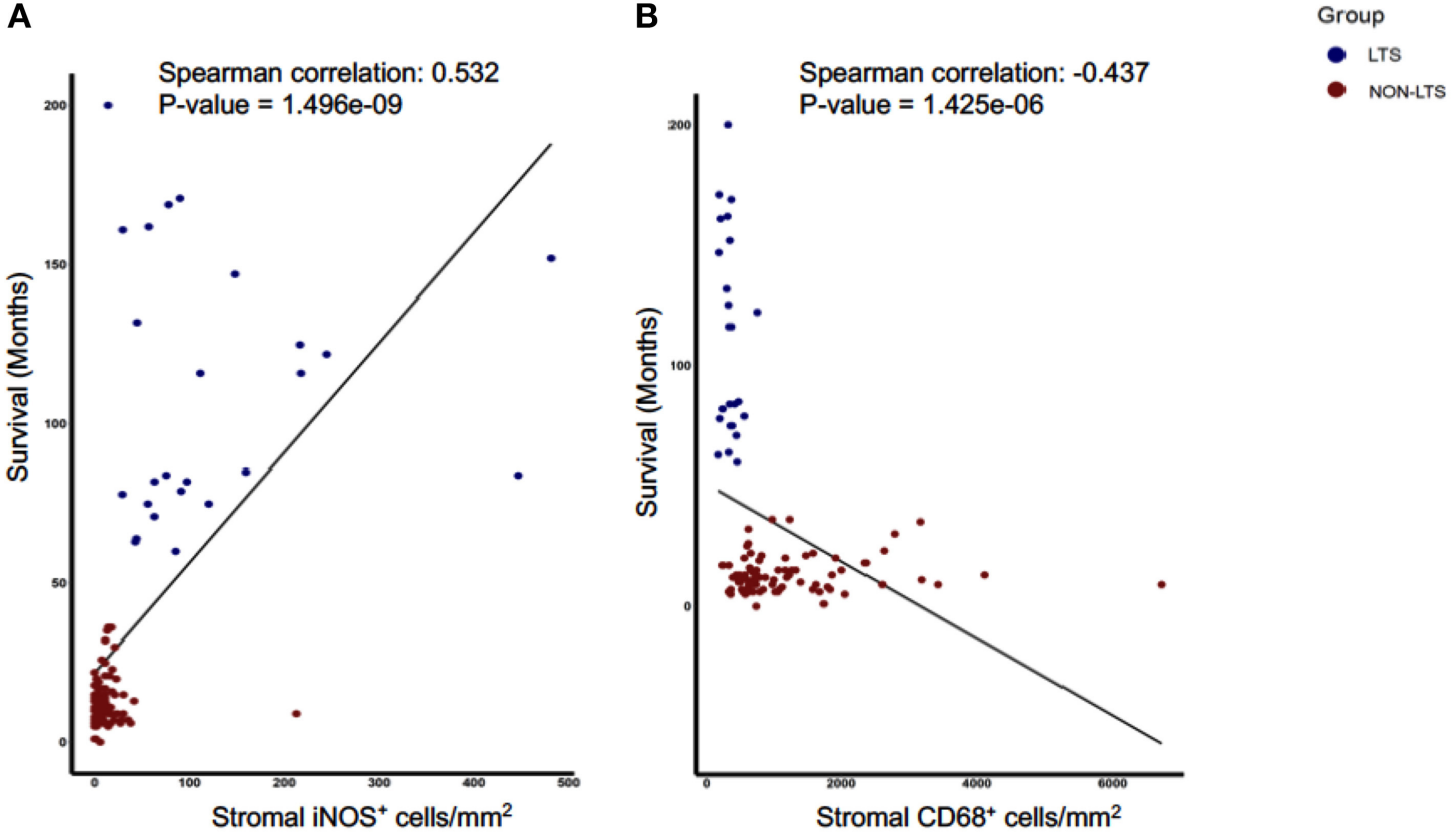
Stromal densities of iNOS and CD68 positive cells display strongest association with survival using Random Forest variable selection. Scatterplots demonstrating association between stromal density and survival for **(A)** iNOS^+^ cells, and **(B)** CD68^+^ cells. Each symbol represents an individual patient. Both the features are significantly (*P* < 0.05) associated with OS (months). Spearman correlation and *P*-values are plotted.

Next, we sought to identify using machine learning, the immune variable (IT or Stromal) that best classifies patients as LTS or NON-LTS and thereby, predicts patient survival. Random-forest (RF) is a decision tree-based machine learning method which uses a bootstrapping method to generate at random, training and testing samples from the dataset (28). Variable selection was performed through the varSelRF package in R, which uses recursive feature elimination to find a minimal set of variables with the best predictive performance (28). Using this approach, we determined that stromal densities of iNOS^+^ and CD68^+^ cells are the two best discriminatory variables for patient survival. Stromal iNOS^+^ leukocytes display a potent positive correlation with OS (Figure 7A) while stromal CD68^+^ (total) macrophages show a strong negative correlation with OS (Figure 7B). The ROC (Receiver Operating Characteristic) curves and ROC values for the final two variables determined by RF selection (i.e., iNOS and CD68) are plotted in Supplementary Figure 7. These findings suggest that iNOS^+^ leukocytes and CD68^+^ macrophages are highly relevant prognostic biomarkers in PDAC patients.

### *In silico* Examination of Survival Associated Features in the PDAC TME

In order to validate our findings in an external cohort, we performed analyses of transcriptional profiles of PDAC tissue in a publicly available dataset first described in a report by Puleo et al. (E-MTAB-6134) (12). For *in silico* studies, we examined only those patients with long vs. short-term survival. Selecting cases with extreme phenotypes/outcomes is a routine practice in transcriptomics analyses of cancer patients in order to identify differentially expressed genes or gene sets without confounding from non-extreme samples (33). As such, we used a quintile-based dichotomization for OS (top 20% vs. bottom 20%). Samples from patients in the aforementioned dataset with survival information (*n* = 288) were divided into the following groups; LTS (*n* = 58, median OS of 63.5 months) and STS (*n* = 58, median OS of 7.2 months).

**Figure 8:**
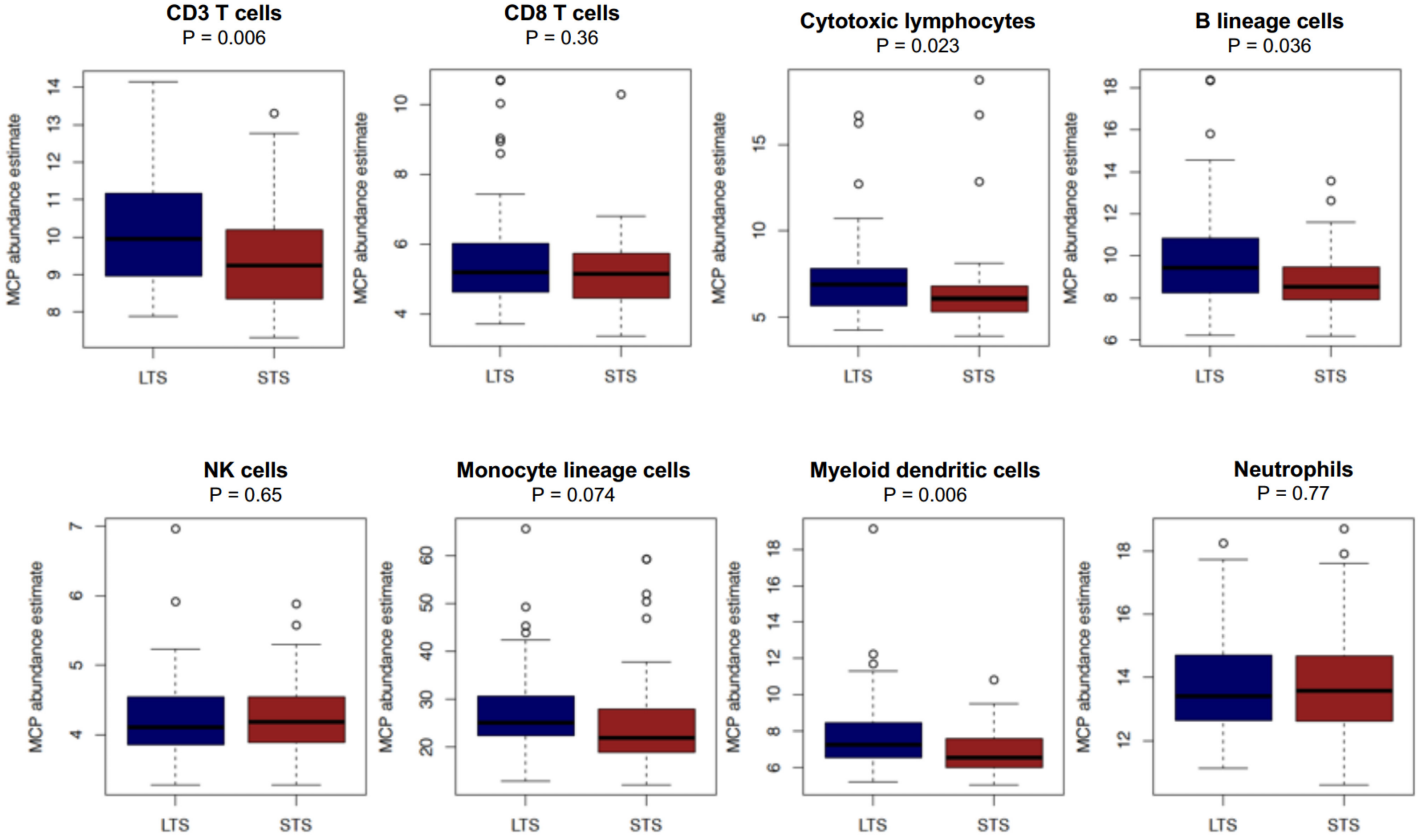
Comparison of immune cell abundance between LTS and STS cases *in silico*. Boxplots demonstrating the distribution of abundance scores for 8 leukocyte subsets as computed by the MCP-Counter method in LTS (*n* = 58) vs. STS (*n* = 58) samples in an external cohort of PDAC cases. The following cell types were quantified CD3^+^ T cells (Pan T cells), CD8^+^ T cells, cytotoxic lymphocytes, B lineage cells, NK cells, monocytic lineage cells, myeloid dendritic cells and neutrophils. Differences between groups were analyzed using the Mann-Whitney *U* test.

First, we performed immune cell profiling in LTS and STS patients *in silico*. Recently, a number of algorithms have been devised to perform cell-type specific deconvolution of bulk transcriptomic (microarray or RNAseq) data (34). We used the Microenvironment Cell Populations-counter (MCP-counter) method developed by Becht et al., which can compute the abundance of eight immune and two non-immune stromal cell subtypes using transcriptional profiles of a bulk tumor sample (35). A comparison of the eight immune cells between LTS and STS-cases revealed an immune landscape comparable to LTS patients in our own cohort (Figure 8). While there were no statistically significant differences between both survival groups in the estimated abundance of CD8^+^ T cells (*P* = 0.36), that of CD3^+^ T cells and B lineage cells was significantly higher in LTS vs. STS-cases (*P* = 0.006 and 0.036, respectively). Furthermore, cytotoxic lymphocyte scores, a functionally defined subset which accounts for mRNA expression from both T cells and NK cells (35), were found to be significantly elevated in LTS cases compared to STS-patients (*P* = 0.023). When compared to STS-patients, the LTS group exhibited no significant differences in the abundance of NK cells (*P* = 0.65), monocyte-lineage cells (*P* = 0.074), and neutrophils (*P* = 0.77). However, myeloid DC abundance scores were markedly higher in LTS compared to STS tumors (*P* = 0.006). Finally, multivariate Cox regression was performed in the cohort including all MCP-Counter populations and available clinical variables (gender, TNM tumor stages, and pathological tumor grades). Only tumor grades and myeloid DC abundance scores were observed to be independently associated with OS in the external cohort (Supplementary Table 4).

Due to the strong association between survival status (LTS vs. NON-LTS) and TLS formation in our cohort, we sought to recapitulate this finding *in silico*. Recently, a 12-gene chemokine signature which could predict TLS presence in colorectal cancer was reported by Coppola et al. in 2011 (25). Using a rank-based sample scoring method for gene signatures (*signscore*) (26), we scored each sample for TLS gene expression. LTS cases were observed to have significantly higher (*P* = 0.016) signature scores for the TLS signature vs. STS cases (Supplementary Figure 8). Collectively, these results provide an independent validation of the presence of TLT formation and dense lymphocyte infiltration in patients who exhibit improved survival in pancreatic cancer.

## Discussion

The distinct TME of PDAC patients poses a significant challenge for treatment with conventional drugs or immunotherapy (15, 16). By examining a clinically rare subset of LTS patients, we sought to determine those features which are highly specific to enhanced survival. Generally, LTS cases exhibit an inactive or inert stroma, and are marked by improved T cell infiltration and a significantly reduced burden of immunosuppressive myeloid cells and Tregs. Furthermore, our findings show evidence for the use of TAM markers as prognostic biomarkers and suggest that therapies targeted to myeloid cells might abolish the uniquely immunosuppressive TME found in pancreatic cancer.

Currently, T cell targeted immunotherapies such as immune checkpoint inhibitors, tumor cell-derived vaccines (e.g., GVAX) and chimeric antigen receptor (CAR)-T cell infusions show only minimal clinical efficacy in PDAC patients (15). Remarkably, although CD8^+^ T cell counts were identified as an independent prognostic factor, no significant differences were observed in CD8^+^ T cell density between LTS and NON-LTS cases in either the intratumoral or the stromal compartment. These findings warrant further elucidation. First, in our cohort, a number of NON-LTS patients exhibiting medium-term survival (between three to five years) contained higher densities of both intratumoral and stromal CD8^+^ T cells. As such, major differences between NON-LTS and LTS cases could not be observed. Secondly, these data suggest that the presence of copious numbers of CD8^+^ T cells alone might not be sufficient to control disease progression in pancreatic cancer. This was corroborated by the seminal work of Balachandran et al. who demonstrated that PDAC patients who were found to have the longest survival displayed the highest number of predicted neoantigens as well as the highest abundance of CD8^+^ T cells but neither variable independently (36).

We found CD4^+^ T cells to be significantly elevated in both the intratumoral and stromal compartments of LTS vs. NON-LTS patients. Furthermore, patients with high CD4^+^ T cell infiltration demonstrated a dramatically improved OS compared to patients with low CD4^+^ T cells, suggesting that patient outcome might be, at least partly, mediated by CD4^+^ helper T cells. Indeed, CD4^+^ T cells are recognized to be essential for tumor immunity as they can exhibit direct cytotoxicity against tumor cells as well as potentiate DC resulting in enhanced CD8^+^ T cell responses (37). The association of CD4^+^ T cell density with OS has been corroborated by a number of other reports (20, 38). However, our report is the first to demonstrate that helper T cells distinguish LTS from NON-LTS cases warranting further investigation into CD4^+^ T cell specific immunotherapies. Evidence for the therapeutic utility of helper T cells is shown in murine models of glioblastoma multiforme where CD4^+^ CAR-T cells were found to perform better than CD8^+^ CAR-T cells, particularly in terms of exhibiting long-term anti-tumor immunity (39). Notably, the immune landscape of LTS cases in our cohort was recapitulated *in silico* in an external cohort of PDAC tissues, where patients in the top quintile (i.e., LTS) of overall survival exhibited higher abundance scores for total (CD3^+^) T cells but not for CD8^+^ T cells compared to STS patients. The MCP-counter algorithm does not specifically estimate the abundance for CD4^+^ T cells but incorporates them into the CD3^+^ T cell category, possibly explaining the higher CD3^+^ T cell scores observed in LTS compared to STS cases (35). We were unable to assess the presence of FOXP3^+^ Tregs *in silico*. Nevertheless, the intratumoral and stromal densities of Tregs was found to be markedly diminished in LTS cases suggesting the presence of a uniquely immunostimulatory TME compared to NON-LTS cases. Intriguingly, MCP-counter derived abundance scores for “cytotoxic lymphocytes” were significantly higher in LTS compared to STS patients. This category is considered to transcriptionally identify T cell and NK cells with cytotoxic ability and suggests the presence of higher numbers of functionally active cytotoxic leukocytes in LTS cases (35). Collectively, these findings indicate that long-term survival in PDAC patients is predicated on both T cell infiltration and the presence of functionally activated T cells. Thus, there is a therapeutic rationale for treating such cases with agonistic antibodies to T cell costimulatory molecules (e.g., CD137, OX40, GITR), a number of which are undergoing clinical trials (40). Such an approach might significantly enhance anti-tumor immunity in PDAC patients with a pre-existing T cell infiltrate.

B cells and DC can prime T cells to target tumor cells due to their capacity for antigen presentation (41, 42). This occurs in secondary lymphoid organs but can also occur in TLT which contain CD20^+^ B cells and DC-LAMP^+^ DC and are considered to be foci of T cell priming (29). We also noted elevated numbers of CD20^+^ B cells and mature DC-LAMP^+^ DC in the stromal compartment of LTS group compared to the NON-LTS group. Most TLTs are found in the invasive margin of the tumor or the stroma and as such, increased densities of stromal B cells and DC in the LTS group are suggestive of the co-localization of these cells within TLT. Further support for this observation was yielded by our *in silico* analyses, where LTS cases exhibited not only higher abundance scores for B lineage cells and myeloid DC but also displayed significantly higher expression of a previously published TLT gene signature compared to STS cases (25). Thus, inducing TLT formation in solid tumors is a potentially promising treatment and a number of agents in preclinical development (e.g., agonistic anti-lymphotoxin beta receptor antibody) warrant further investigation (29).

Finally, the most striking immunological differences between LTS and NON-LTS cases were observed in the myeloid compartment. Our work aligns with the findings of Ino et al., who showed that CD68^+^ and CD163^+^ macrophages were associated with significantly worse OS in PDAC (20). It is therefore plausible why therapies targeted to TAMs and other myeloid cells currently display promise both in pre-clinical and clinical studies of pancreatic cancer (15–17). TAM-targeted therapies involve depletion (e.g., CSF1Ri), interrupting their recruitment to the TME (CCR2i) or repolarizing TAMs to an immunostimulatory (M1) phenotype (e.g., agonist anti-CD40 antibodies) (15–17). Such treatments aim to abrogate TAM-mediated immunosuppression but have also been shown to significantly remodel tumor stroma (43). We observed an inverse relationship between iNOS^+^ leukocytes and CD68^+^ as well as CD163^+^ leukocytes in LTS and NON-LTS cases. It is pertinent to note that while iNOS is considered to be a marker for M1 macrophages (44), it is also expressed by neutrophils (45). As such, we used the term iNOS^+^ leukocytes to describe all immune cells were positive for iNOS. Furthermore, iNOS-mediated NO production in both macrophages and neutrophils can induce direct tumor cytotoxicity (45, 46). Thus, it can be postulated that myeloid cells play an essential role in the improved survival of LTS cases. However, we could not determine differences between LTS and STS patients for myeloid lineage cell abundance scores in the external cohort. This is plausible as cell-type deconvolution methods rely upon pre-defined transcriptional signatures to identify a particular subset and thus, may be unable to distinguish between the heterogeneous subsets of the myeloid lineage (34, 47). Finally, we utilized a Random-Forest based variable selection method to identify a minimum number of variables which could predict survival in our cohort (28). Thus, our findings offer a rationale for the use of either iNOS and/or CD68 as prognostic biomarkers for PDAC. From our results, it can be hypothesized that a higher burden of stromal CD68^+^ or CD163^+^ TAMs might prevent intratumoral infiltration of T cells. Support for this concept is provided by studies in mice where stromal macrophages were found to trap CD8^+^ T cells in the stroma and their depletion with CSF1Ri, led to significantly enhanced infiltration of CD8^+^ T cells into tumor nests (48).

Our study also shows that LTS cases can be distinguished by having a largely inactivated stromal profile thereby suggesting that stromal modulation might extend survival in pancreatic cancer. However, therapeutic manipulation of the stroma in PDAC has shown mixed results in experimental models. Depletion of αSMA^+^ myofibroblasts in autochthonous murine tumors resulted in advanced disease progression with invasive, undifferentiated tumors and increased hypoxia and EMT (49). On the other hand, inhibition of focal adhesion kinase (FAK) in murine models was shown to diminish fibrosis resulting in a notable reduction in intratumoral macrophages and Tregs (50). In addition, studies have also shown that activated pancreatic stellate cells (i.e., CAFs) can limit T cell infiltration into the intratumoral compartment (51). Indeed, in our study we noted that diminished fibrosis was associated with reduced numbers of immunosuppressive and increased numbers of immunostimulatory leukocytes, as nearly half of the LTS patients (42%) displayed an “inert” stromal subtype. Our results are also corroborated by the work of Mahajan et al., who also reported immune cell differences between stromal subtypes (52). The authors showed that while fibrolytic (αSMA^high^/collagen^low^) stromal subtype displays a higher abundance of CD206^+^ macrophages (which are also deemed to be M2 macrophages) and reduced CD8^+^ T cells, fibrogenic (αSMA^high^/collagen^high^) stroma are marked by increased CD8^+^ T cells and CD68^+^ macrophages (52). In our study, it was shown that 57% of the NON-LTS group displayed fibrolytic and fibrogenic stromal subtypes and while these cases contained comparable levels of CD8^+^ T cells to LTS cases, they also exhibited a notably higher burden of CD163^+^ (M2) and CD68^+^ TAMs. Thus, our study supports the concept of stromal-immune crosstalk with respect to the myeloid compartment. Interfering with this crosstalk might limit stromal activation and TAM-mediated immunosuppression. This was shown recently by an elegant study in an autochthonous murine model of PDAC, where the authors disrupted pancreatic stellate cell activation and ECM deposition using Halifuginone, an analog of febrifugine (53). Inhibition of fibrosis with Halifuginone in mice resulted in a marked infiltration of iNOS^+^ leukocytes and CD8^+^ T cells (53). Thus, such an approach might be a viable strategy for modulating the stroma and repolarizing myeloid cells to an immunostimulatory phenotype.

A limitation of our study is the low number of cases in the LTS group. Nevertheless, our results provide substantial evidence that long-term survival in pancreatic cancer is associated with distinct immune and stromal profiles suggesting the use of combinatorial therapy to improve treatment outcomes in PDAC. NGS mutational profiling revealed no major differences between LTS and NON-LTS cases and furthermore, it is known that the four primary driver mutations in PDAC currently remain unactionable targets. Thus, while further efforts are required to comprehensively phenotype the immunological and stromal cell diversity in pancreatic cancer, our approach highlights a number of immune markers for use as prognostic tools or as drug targets for combinatorial immunotherapies.

## Data Availability Statement

The datasets presented in this study can be found in online repositories. The names of the repository/repositories and accession number(s) can be found in the article or Supplementary Material.

## Ethics Statement

The studies involving human participants were reviewed and approved by Ethics Committee of the Canton of Bern (KEK Nr 200/14). The patients/participants provided their written informed consent to participate in this study.

## Author Contributions

EK conceived and supervised the study. EK and HS devised all analyses and wrote the manuscript. HS, AA, and SE-C performed statistical analyses and interpreted data. AA revised the manuscript. TG and HS performed *in silico* analyses and interpreted data. MS provided input on interpretation of the data. BG provided the clinical information. All authors contributed to the article and approved the submitted version.

## Conflict of Interest

The authors declare that the research was conducted in the absence of any commercial or financial relationships that could be construed as a potential conflict of interest.
