# Supplementary material for: Distinct Stromal and Immune Features Collectively Contribute to Long-Term Survival in Pancreatic Cancer
Source: Front Immunol. 2021 Feb 19;12:643529. doi: 10.3389/fimmu.2021.643529 (PMC7933000; doi:10.3389/fimmu.2021.643529)
Supplement: Supplementary file 1 [file Data_Sheet_1.docx]

**Supplementary Information for Sadozai et al. 2021. Frontiers in Immunology.**

**Supplementary Materials and Methods**

**Patients and tissues**

112 well-characterized, surgically resected PDACs, stage I-III, including a cohort of 25 long-term survivors (LTS, OS ≥ 60 months) were available for this study. Patients were selected based on tissue availability and accessibility to full follow-up information. All cases were reviewed concerning histomorphologic data by using representative whole-tissue hematoxylin and eosin (H&E) stained slides, while clinical data were obtained from corresponding reports. Clinicopathologic information for all patients included age, gender, tumor diameter, tumor grade, number of positive lymph nodes, total number of lymph nodes harvested, pTNM stage, perineural, blood vessel and lymphatic invasion, resection margin status, as well as overall (OS) and progression-free survival (PFS). Tumors from patients treated with neoadjuvant therapy, stage IV tumors, as well as carcinomas with histology other than ductal adenocarcinoma (such as acinar cell carcinomas, adenosquamous carcinomas, neuroendocrine carcinomas, mixed subtypes and mucinous neoplasms with associated invasive carcinoma) were excluded from the study. Staging was performed using the AJCC 8th edition (1). The study was approved by the Ethics committee of the Canton of Bern (KEK Nr 200/14).

***Next-generation Tissue Microarray (ngTMA) construction***

Sequencing data are available from 25 LTS- and 78 NON-LTS cases. Tissue microarrays were constructed using the ngTMA^®^ approach (2). For each patient, one hematoxylin and eosin (H&E) stained representative whole tissue slide was scanned (Panoramic P250, 3DHistech, Budapest, Hungary). Using a tissue microarray annotation tool of 0.6 mm in diameter, slides were digitally annotated and punches from 8 different tumor regions to account for tumor heterogeneity were included. Next, corresponding formalin-fixed (10% buffered formalin) paraffin-embedded tissue blocks were loaded into an automated tissue microarrayer (TMA Grandmaster, 3DHistech, Budapest, Hungary). The digital slides were aligned with the corresponding donor block. Annotated regions were cored from the donor block and transferred to the recipient ngTMA.

***NGS Sequencing using Ion Ampliseq^TM^ Hotspot Cancer Panel v2 (ThermoFisher).***

The genes included in the Hotspot Cancer Panel v2 are depicted in the table below and a full range of covered mutations are available on the vendor’s home page (<https://www.thermofisher.com/order/catalog/product/4475346#/4475346>).


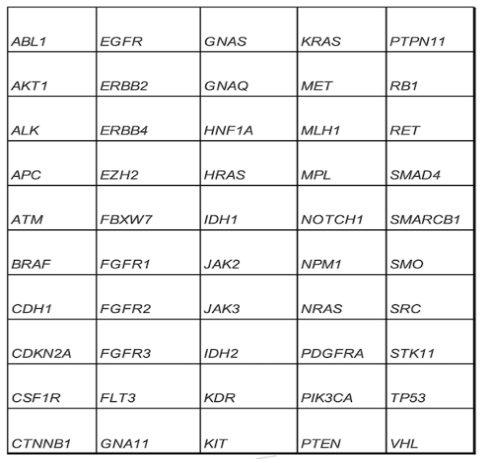


*Libraries preparation for targeted NGS with the IonTorrent Platform*

The Ion AmpliSeq™ Cancer Hotspot Panel v2 panel was designed to allow amplification-based capture and sequencing of mutational hotspot regions of 50 cancer-related genes. This panel includes 207 primer pairs and requires a minimum initial amount of 10 ng of DNA as input. After DNA extraction and quantification, multiplex PCR for targets enrichment was performed using genomic DNA mixed with primer pools and the Ion AmpliSeq™ HiFi master mix (Ion AmpliSeq™ Library Kit Plus, Thermo Fisher Scientific) for 2 min at 99°C, followed by 22 cycles of 99°C for 15 sec and 60°C for 4 min and holding at 10°C and libraries were prepared according to the manufacturer’s recommendations. The library concentration was determined using an Ion Universal Library Quantitation Kit (Thermo Fisher Scientific). 40 pM for each library were loaded into the IonChef^TM^ System using the IC Hi-Q sequencing Kit (Thermo Fisher Scientific) for fully automated emulsion polymerase chain reaction (PCR) and chip loading (32 samples were multiplexed on one 530 chip). Finally, loaded chips were sequenced (500 flows) using the Ion S5 Sequencing Kit using the S5 Sequencer (Thermo Fisher Scientific).

***Sequencing data analysis***

Raw data (FASTQ files) for each sample were processed for the alignment of sequencing reads with the human genome reference (hg19) using the Torrent Suite software v5.2 (Thermo Fisher Scientific). The alignment pipeline also included signaling processing, base calling, quality score assignment, adapter trimming and control of mapping quality. Coverage metrics for each amplicon (minimal acceptable coverage threshold was set at 500x) was obtained by running the Coverage Analysis Plugin software v5.6.0(Thermo Fisher Scientific). Base calling was performed using the IonReporter v5.6 (Thermo Fisher Scientific). We choose these specific mutations caller strategy as it allows to identify somatic mutations down to 5% mutant allele fraction (MAF) (i.e. 5% of the reads harboring a given mutation). Finally, all candidate mutations were manually reviewed using the Integrative Genomics Viewer (3).

***Assessment of tumor budding and of gland-forming component***

Tumor budding has been previously described (4) and was deﬁned as single tumor cells or tumor cell clusters of up to four cells. Whole tissue sections of the PDACs, stained with H&E as in routine diagnostics, were utilized. Briefly, tumor buds were counted in one hotspot area at 20x magnification (field area 950 μm^2^) and the number of tumour buds was divided by 1.21 to obtain the number of buds in an area of 785 μm^2^. Density of tumor buds was assigned into three groups: low budding (BD-1): 0-4 buds; intermediate budding (BD-2): 5-9 buds; and high budding (BD-3): ≥ 10 buds.

The gland-forming component was estimated using H&E stained whole tissue slides as percentage of the whole tumor area.

REFERENCES

1. Kakar S, Pawlik TM, Allen PJ, Vauthey J-N. Exocrine Pancreas. AJCC Cancer Staging Man. Springer International Publishing; 2016. page 337–47.
2. Zlobec I, Suter G, Perren A, Lugli A.: A Next-generation Tissue Microarray (ngTMA) Protocol for Biomarker Studies. J Vis Exp (2014) (91):51893. doi: 10.3791/51893.
3. Thorvaldsdóttir H, Robinson JT, Mesirov JP : Integrative Genomics Viewer (IGV): high-performance genomics data visualization and exploration. Brief Bioinform (2013) 14(2):178-92.
4. Karamitopoulou E, Wartenberg M, Zlobec I, Cibin S, Worni M, Gloor B, et al.: Tumour budding in pancreatic cancer revisited: validation of the ITBCC scoring system. Histopathology (2018) 73(1):137-46.

**Supplementary Figure Legends**

**Supplementary Figure 1: Overview of study design.**

**Supplementary Figure 2:** **Representative images for TLT.**

**(A):** Representative images (x150) of a TLT structure in PDAC stained with H&E, anti-CD3 and anti-CD20 antibodies. **(B):** Kaplan-Meier curves showing association between presence of TLTs and OS.

**Supplementary Figure 3: Representative images for tumor budding and gland forming component:**

Representative cases (H&E, x150) displaying high gland-forming component and low-grade tumor budding **(A)** vs. low gland-forming component and high-grade tumor budding **(B).** Arrow: tumor buds.

**Supplementary Figure 4: Representative images for stromal subtyping.** Representative cases depicting **(a)** inert, **(b)** desmoplastic, **(c)** fibrolytic and **(d)** fibrogenic stromal subtypes (x150). Stromal subtyping was performed using the following stains; αSMA (red chromogen), Pan-cytokeratin (brown chromogen) and collagen (Masson`s Trichrome).

**Supplementary Figure 5.** Representative images from LTS and NON-LTS cases (x200). A selection of representative immunohistochemistry stains is presented with Pan-cytokeratin (brown chromogen) and CD3, CD4, CD8 (red chromogen) representing lymphocyte subsets as well as iNOS, CD163 and CD68 (red chromogen) representing myeloid cell populations.

**Supplementary Figure 6: Stromal leukocyte densities and association with survival of PDAC cases.**

Kaplan-Meier curves comparing the OS of PDAC cases with high (red) and low (blue) stromal densities of tumor infiltrating lymphocytes (CD3^+^ T cells, CD4^+^ T cells, CD8^+^ T cells, CD20^+^ B cells and FOXP3^+^ Tregs), myeloid cell populations (CD68^+^ macrophages, CD163^+^ macrophages, iNOS^+^ myeloid cells) and DC-LAMP^+^ dendritic cells. Cases were dichotomized as high or low for each immune cell density via a median cut-off. Statistical comparisons were performed using the log-rank test.

**Supplementary Figure 7: Random Forest Modeling ROC curves.**

The top two selected variables (stromal iNOS and CD68 density) for classification of LTS and NON-LTS cases and their corresponding AUC value is shown.

**Supplementary Figure 8: Comparison of TLS gene signature between survival subgroups.**

Boxplots displaying the distribution of scores for a TLS-associated chemokine gene signature. The *singscore* package was used to determine the TLS gene signature score for each sample (see methods). Differences between LTS (*n*=58) vs. STS (*n*=58) groups were analyzed using the Mann-Whitney U test

**Supplementary Tables**

**Supplementary Table 1: Clinical and pathological features of the cohort.**

Quantitative data presented as group medians with interquartile range (IQR) in brackets. Categorical data are shown as number of cases and as a percentage of survival subgroups in brackets. Bolded values denote significant results. Statistical comparisons were performed using Mann-Whitney U Test and Chi-Square test. Bolded values denote significant results.

| **Parameter** | **LTS (*n* = 25)** | **NON-LTS (*n* = 87)** | **P Value** |
| --- | --- | --- | --- |
| **OS (months)** | 85 (60-200) | 12 (3-36) | **<0.001** |
| **PFS (months)** | 84 (60-196) | 6 (3-29) | **<0.001** |
| **Sex** |  |  | 0.7 |
| *F* | 14 (56%) | 40 (48%) |  |
| *M* | 11 (44%) | 44 (52%) |  |
| **Age (years)** | 69 (61-75) | 65 (59-71) | 0.2 |
| **Size (mm)** | 30 (25-30) | 30 (25-40) | 0.3 |
| **CA19-9 (U/ml)** | 159 (35-800) | 380 (30-6835) | **0.01** |
| **Anatomic site** |  |  | 0.56 |
| Head | 22 (88%) | 70 (83%) |  |
| All others | 3 (12%) | 14 (17%) |  |
| **Grade** |  |  | **<0.001** |
| *1* | 14 (56%) | 11 (13%) |  |
| *2* | 3 (12%) | 41 (49%) |  |
| *3* | 8 (32%) | 32 (38%) |  |
| **UICC Stage 8^th^ Edition** |  |  | **0.008** |
| *IA* | 1 (4.0%) | 6 (7%) |  |
| *IB* | 10 (40%) | 5 (6%) |  |
| *IIA* | 1 (4.0%) | 4 (5%) |  |
| *IIB* | 11 (44%) | 49 (56%) |  |
| *III* | 2 (8.0%) | 23 (26%) |  |

**Supplementary Table 2: PDAC driver gene mutations (*KRAS*, *TP5*3, *CDK2NA* and *SMAD4*) across LTS and NON-LTS groups.**

Data are shown number of cases per group (and as percentage of survival subgroup in brackets). Statistical comparisons were performed using Fisher’s Exact test.

|  | **LTS,** *n* **= 25** | **NON-LTS,** *n* **= 78** | **P Value** |
| --- | --- | --- | --- |
| ***KRAS*** |  |  | >0.99 |
| MUT | 24 (96%) | 73 (94%) |  |
| WT | 1 (4%) | 5 (6%) |  |
| ***TP53*** |  |  | 0.80 |
| MUT | 17 (68%) | 56 (72%) |  |
| WT | 8 (32%) | 22 (28%) |  |
| ***CDK2NA*** |  |  | 0.15 |
| MUT | 2 (8%) | 18 (23%) |  |
| WT | 23 (92%) | 60 (77%) |  |
| ***SMAD4*** |  |  | 0.51 |
| MUT | 2 (8%) | 12 (15%) |  |
| WT | 23 (92%) | 66 (85%) |  |

**Supplementary Table 3. Multivariate cox regression of all primary features in the present (University of Bern) cohort.**

Model performance test is performed at the end of the table. Bolded values in the last column denote significant results.

| **Parameters** | **HR** | **lower .95** | **upper .95** | **P value** |
| --- | --- | --- | --- | --- |
| CD3 S | 1.001003 | 1.00012 | 1.001886 | **0.02595** |
| CD4 S | 0.998119 | 0.996678 | 0.999561 | **0.01061** |
| CD8 S | 0.997823 | 0.996611 | 0.999038 | **0.000445** |
| CD20 S | 1.001441 | 1.00033 | 1.002554 | **0.011042** |
| CD68 S | 1.000388 | 0.999826 | 1.000951 | 0.176025 |
| CD163 S | 0.99997 | 0.999519 | 1.000421 | 0.896196 |
| iNOS S | 0.998358 | 0.991358 | 1.005407 | 0.647045 |
| DC-LAMP S | 0.995403 | 0.991113 | 0.999712 | **0.036538** |
| FOXP3 S | 1.00403 | 1.000643 | 1.007428 | **0.019664** |
| CD3 IT | 1.008021 | 0.981338 | 1.035429 | 0.559449 |
| CD4 IT | 0.940064 | 0.86351 | 1.023405 | 0.15383 |
| CD8 IT | 1.018852 | 0.996444 | 1.041764 | 0.099756 |
| CD20 IT | 0.976266 | 0.614782 | 1.550298 | 0.918917 |
| CD68 IT | 0.992323 | 0.965294 | 1.020109 | 0.58442 |
| CD163 IT | 1.015146 | 1.003463 | 1.026965 | **0.010918** |
| iNOS IT | 0.987654 | 0.963422 | 1.012494 | 0.326973 |
| DC-LAMP IT | 1.015528 | 0.95285 | 1.082329 | 0.635462 |
| FOXP3 IT | 0.994812 | 0.964219 | 1.026376 | 0.744138 |
| Sex | 0.632056 | 0.39173 | 1.01982 | 0.060168 |
| Age | 0.974742 | 0.952607 | 0.997392 | **0.029054** |
| Size | 1.004739 | 0.987302 | 1.022483 | 0.596622 |
| UICCIB | 4.029215 | 1.037952 | 15.64096 | **0.044031** |
| UICCIIA | 7.794558 | 1.414953 | 42.9379 | **0.018341** |
| UICCIIB | 7.257432 | 2.040314 | 25.81482 | **0.002203** |
| UICCIII | 11.08317 | 2.72406 | 45.09324 | **0.000781** |
|  |  |  |  |  |
| Model Performance | |  |  |  |
|  | P value |  |  |  |
| logtest | 2.56E-08 |  |  |  |
| waldtest | 1.22E-06 |  |  |  |

**Supplementary Table 4. Multivariate cox regression of all primary features in the cohort of Puleo et al. (E-MTAB-6134)**

Model performance test is performed at the end of the table. Bolded values in the last column denote significant results.

| Parameters | HR | lower .95 | upper .95 | | P value |
| --- | --- | --- | --- | --- | --- |
| Sex | 1.30231 | 0.939564797 | 1.805104 | 0.112802 | |
| Tumor grading G2 | 1.176222 | 0.832679906 | 1.6615 | 0.357064 | |
| Tumor grading G3 | 1.725331 | 1.098394689 | 2.710108 | **0.017918** | |
| TNM tumor grading T1N1 | 2.452802 | 0.456499412 | 13.17907 | 0.295617 | |
| TNM tumor grading T2N0 | 2.176556 | 0.552832486 | 8.569315 | 0.266008 | |
| TNM tumor grading T2N1 | 2.778443 | 0.788730082 | 9.787565 | 0.111708 | |
| TNM tumor grading T3N0 | 1.59364 | 0.468374248 | 5.422349 | 0.455716 | |
| TNM tumor grading T3N1 | 3.057568 | 0.929650939 | 10.05616 | 0.065786 | |
| TLS Score | 0.425543 | 0.024061106 | 7.526128 | 0.559953 | |
| T cell | 0.952762 | 0.831755721 | 1.091373 | 0.485012 | |
| T cell CD8 | 1.115386 | 0.962168686 | 1.293003 | 0.147496 | |
| Cytotoxic lymphocytes | 0.97402 | 0.896816917 | 1.057869 | 0.532125 | |
| NK cell | 1.069511 | 0.803436681 | 1.423702 | 0.6452 | |
| B cell | 1.026859 | 0.974107279 | 1.082467 | 0.32462 | |
| Monocyte | 1.01213 | 0.990167305 | 1.034581 | 0.281399 | |
| Myeloid dendritic cell | 0.830558 | 0.734229071 | 0.939525 | **0.00316** | |
| Neutrophil | 1.016417 | 0.934392135 | 1.105642 | 0.704466 | |
| Endothelial cell | 1.025056 | 0.951841727 | 1.103903 | 0.512761 | |
| Cancer associated fibroblast | 1.000187 | 0.999028974 | 1.001347 | 0.75157 | |
|  |  |  |  |  | |
| **Model performance** | |  |  |  | |
|  | P value |  |  |  | |
| logtest | 0.006548 |  |  |  | |
| waldtest | 0.018285 |  |  |  | |
