# Supplementary figures and images for: Distinct Stromal and Immune Features Collectively Contribute to Long-Term Survival in Pancreatic Cancer
Source: Front Immunol. 2021 Feb 19;12:643529. doi: 10.3389/fimmu.2021.643529 (PMC7933000; doi:10.3389/fimmu.2021.643529)

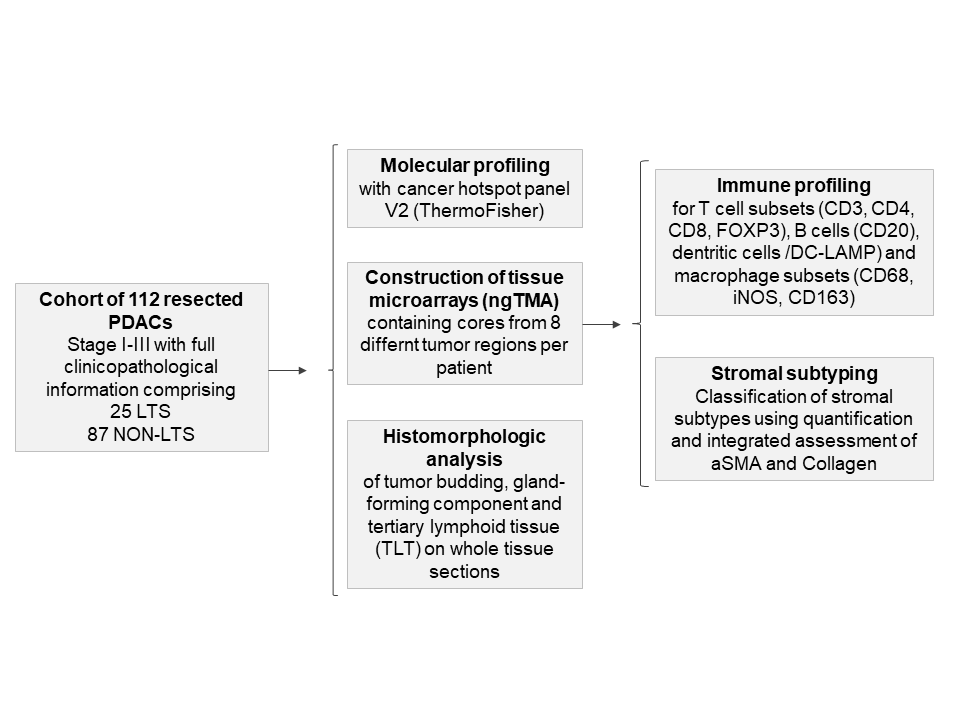

Supplement: Supplementary file 2 [file Image_1.TIF]

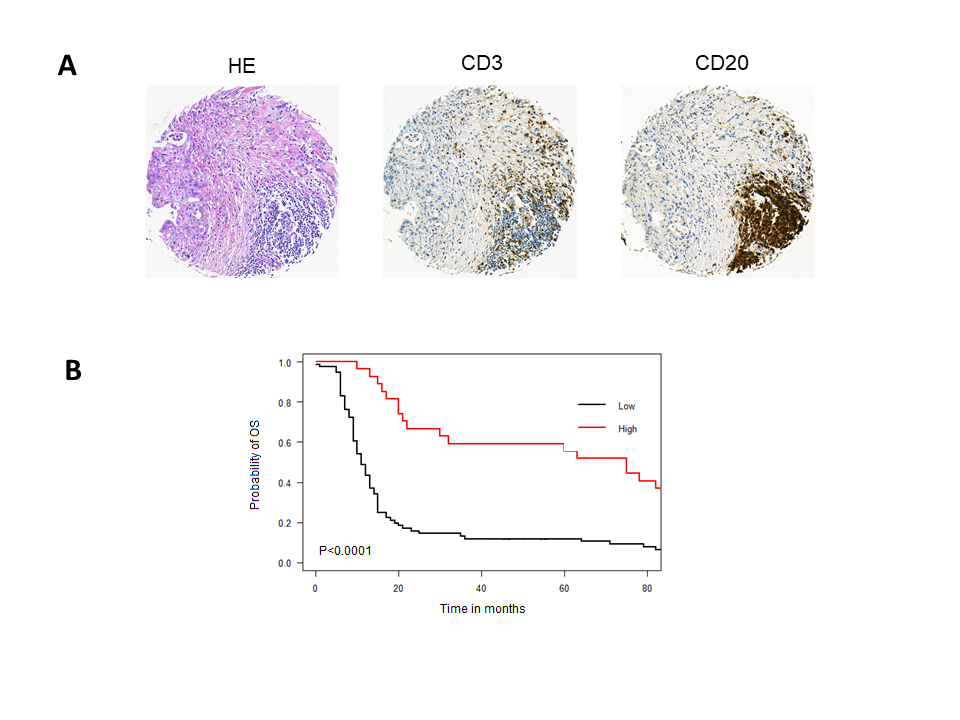

Supplement: Supplementary file 3 [file Image_2.TIF]

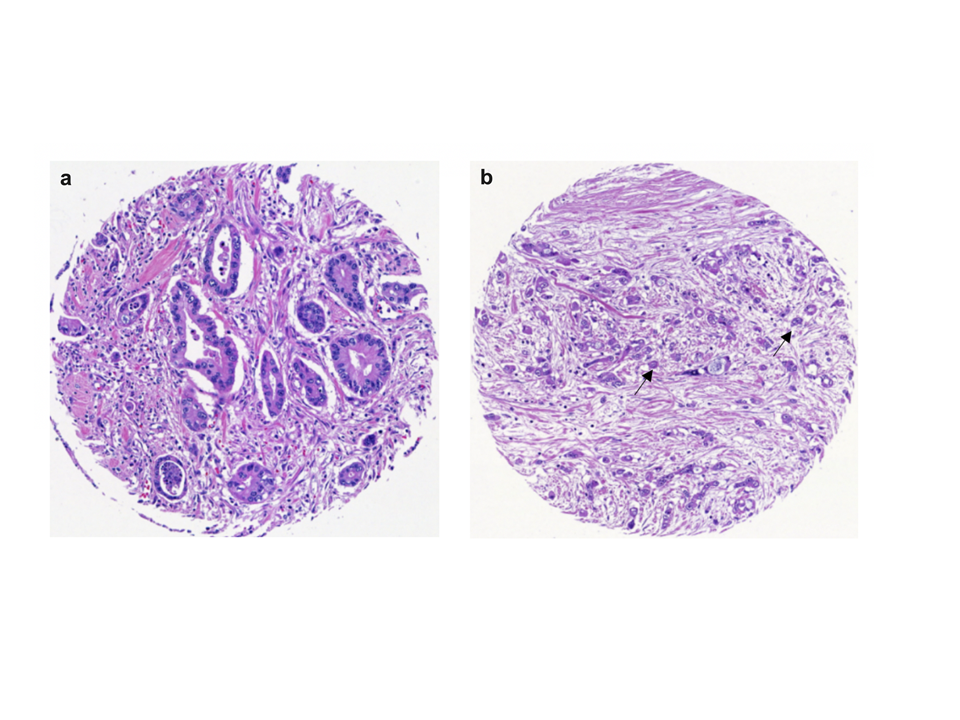

Supplement: Supplementary file 4 [file Image_3.TIF]

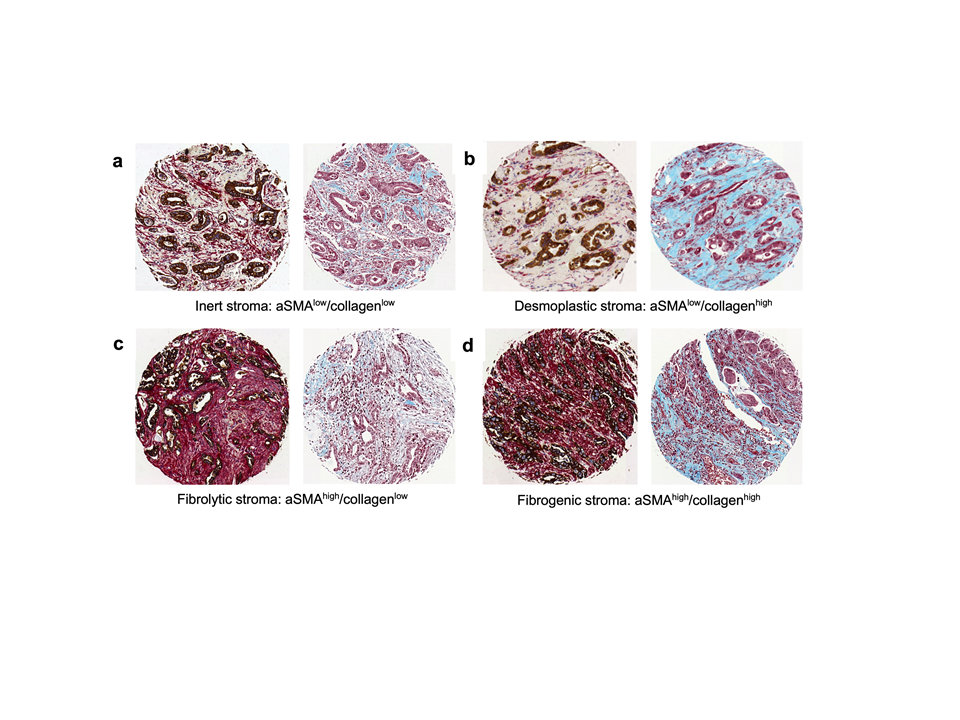

Supplement: Supplementary file 5 [file Image_4.TIF]

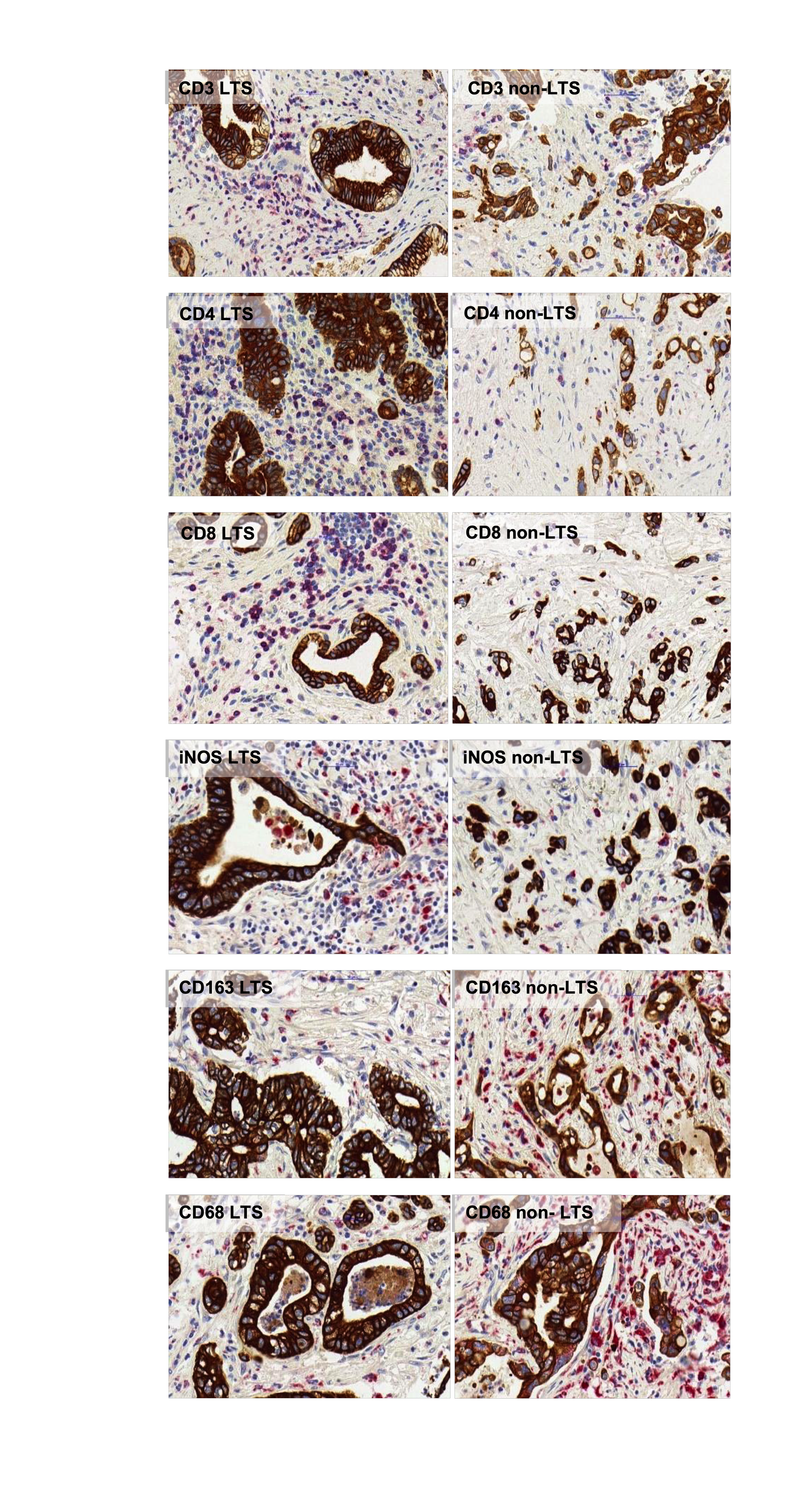

Supplement: Supplementary file 6 [file Image_5.TIFF]

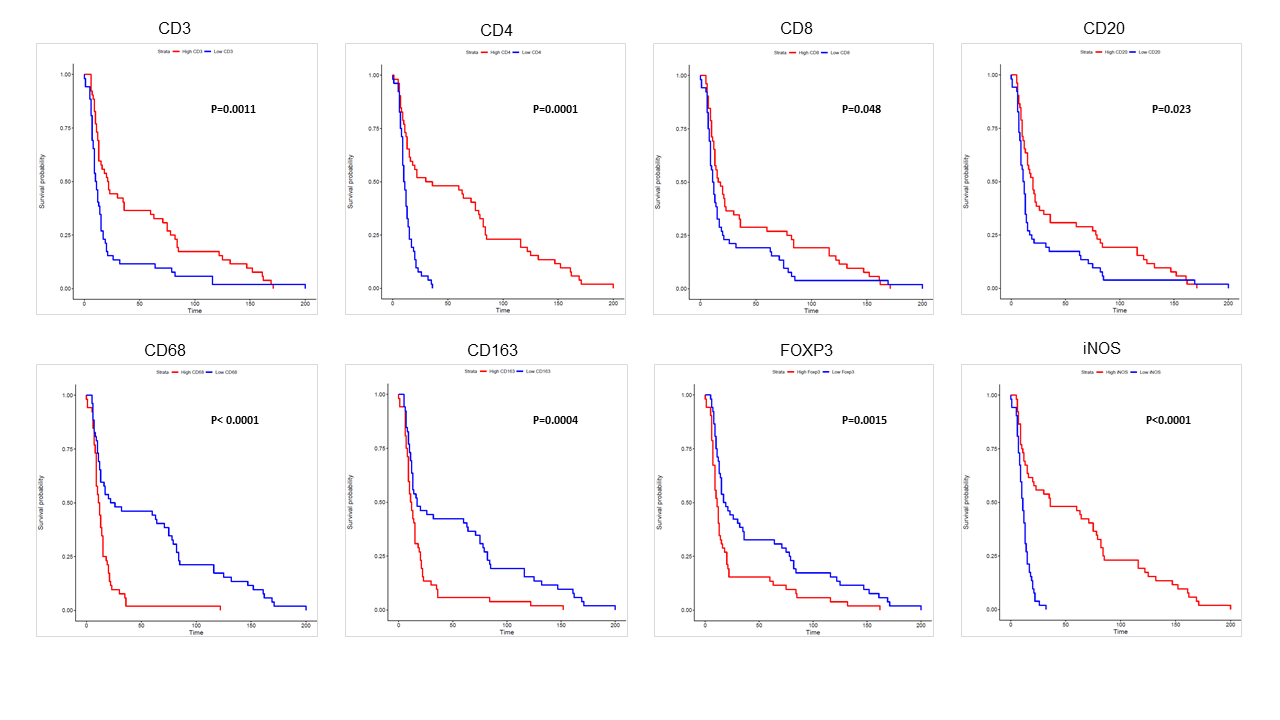

Supplement: Supplementary file 7 [file Image_6.TIF]

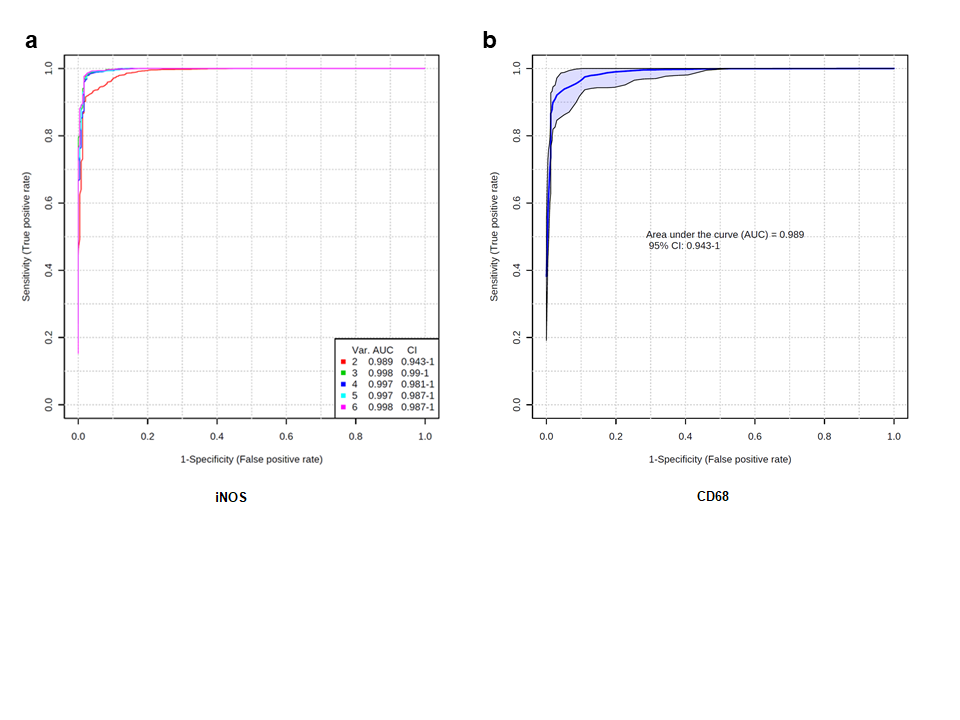

Supplement: Supplementary file 8 [file Image_7.TIF]

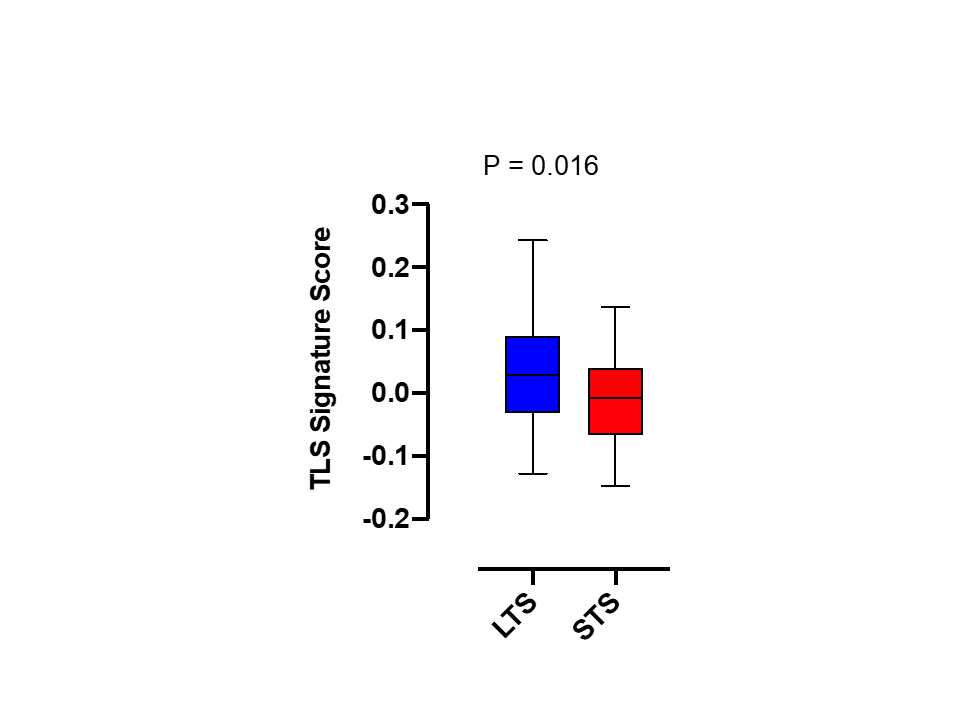

Supplement: Supplementary file 9 [file Image_8.TIF]
